# NPS detection in prison: A systematic literature review of use, drug form, and analytical approaches

**DOI:** 10.1002/dta.3263

**Published:** 2022-04-20

**Authors:** Giorgia Vaccaro, Anna Massariol, Amira Guirguis, Stewart B. Kirton, Jacqueline L. Stair

**Affiliations:** ^1^ Department of Clinical, Pharmaceutical and Biological Sciences, School of Life and Medical Sciences University of Hertfordshire Hatfield UK; ^2^ Swansea University Medical School, The Grove, Singleton Campus Swansea UK

**Keywords:** new psychoactive substances, NPS, prisons, synthetic cannabinoids, systematic literature review

## Abstract

This paper presents a systematic literature review on the detection of new psychoactive substances (NPS) in prison settings. It includes the most frequently reported NPS classes, the routes and forms used for smuggling, and the methods employed to analyse biological and non‐biological samples. The search was carried out using MEDLINE (EBSCO), Scopus (ELSEVIER), PubMed (NCBI), and Web of Science (Clarivate) databases, along with reports from the grey literature in line with the PRISMA‐S guidelines. A total of 2708 records were identified, of which 50 met the inclusion criteria. Findings showed the most prevalent NPS class reported in prison was synthetic cannabinoids (SCs). The most frequently reported SCs in non‐biological samples were 4F‐MDMB‐BINACA, MDMB‐4en‐PINACA, and 5F‐ADB. These were smuggled mainly through the postal services deposited on paper or herbal matrices. Concentrations of SCs detected on seized paper ranged between 0.05 and 1.17 mg/cm^2^. The SCs most frequently reported in biological specimens (i.e., urine, blood, saliva, and wastewater) were 5F‐MDMB‐PICA, 4F‐MDMB‐BINACA, and MDMB‐4en‐PINACA. Concentrations of SCs reported in femoral blood and serum were 0.12–0.48 ng/ml and 34–17 ng/ml, respectively. Hyphenated techniques were predominantly employed and generally successful for the detection of NPS in biological (i.e., LC‐HRMS/MS) and non‐biological samples (i.e., LC‐HRMS/MS and GC–MS). The onsite technique IMS showed promise for detecting SCs in various forms; however, immunoassays were not recommended. Future work should focus on accurate in‐field detection of SCs deposited on paper and in urine and saliva to improve real‐time decision‐making, as well as wastewater and air monitoring for overall drug use trends.

## Graphical abstract

The most predominant classes of NPS that have been reported in prison settings, the routes and forms through which they are smuggled into prison, and analytical methods employed to analyse prison‐related biological and non‐biological samples are critically reviewed.

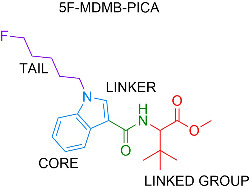

## 1 INTRODUCTION

In recent years, the use of new psychoactive substances (NPS) in prison settings has become a cause of concern internationally.^1^, ^2^, ^3^, ^4^, ^5^, ^6^, ^7^, ^8^, ^9^ The situation reported by 24 countries including the United Kingdom, Germany, Sweden, Hungary, Latvia, Australia, and the United States^9^, ^10^, ^11^ has proven particularly challenging. It has been reported that the use of NPS in prisons has led to increased levels of violence, organized crime, bullying, aggression, and debt.^8^, ^9^, ^10^, ^12^ Although initial measures including training modules for staff, implementation of mandatory drug testing (MDT), infrastructural changes, and/or legislative restrictions,^9^ NPS use in prison remains an issue of major concern.^13^ Whilst there is evidence suggesting that the use of NPS worldwide may be declining, this trend is not observed in marginalized groups, including prison populations.^14^ Use has increased among such populations, for instance, seizures of NPS in UK prisons have increased from 4560 in 2017 to 9114 in 2021.^15^ Thus, timely and collated information focused on the identification of NPS in the prison environment is critical to further understand and ultimately tackle NPS use in this setting.

NPS are defined by the United Nation Office on Drugs and Crime (UNODC) and European Monitoring Centre on Drugs and Drugs Abuse (EMCDDA) as “substances of abuse, either in a pure form or a preparation, that are not controlled by the 1961 Single Convention on Narcotic Drugs or the 1971 Convention on Psychotropic Substances, but which may pose a public health threat.”^1^, ^16^ In addition, NPS have been associated with public health risks similar to traditional drugs of abuse (TdA), and they have also been shown to induce unpredictable health risks. The World Drug Report 2020 further specifies that “the term ‘new’ does not necessarily refer to new inventions, but to substances that have recently become available.”^16^ Due to the structural diversity of NPS, they are largely classified according to their substance groups, for example, aminoindanes, phencyclidine‐type substances, phenethylamines, piperazines, plant‐based substances, synthetic cannabinoids (SCs), synthetic cathinones, tryptamines, and “other” substances such as designer opioids and benzodiazepines.^16^

The use of NPS in prisons was first reported in the United Kingdom around 2013^17^ and in the years to follow in other European and non‐European countries.^9^ These compounds represented a valid alternative to TdA because of their low price, ease of availability, and undetectability.^2^, ^3^, ^7^, ^8^, ^9^, ^10^ In addition, high potency NPS, for example, SCs, are popular amongst prisoners as the desired effect can be achieved with a lesser amount of substance and hence for a cheaper price.^1^, ^8^, ^18^ In particular, SCs are used in this environment to aid in coping with imprisonment, sustaining existing habits, and for self‐medication or pleasure.^6^ Until a few years ago NPS in the United Kingdom were not normally screened in routine MDT,^10^ making them an attractive alternative to TdA. Despite that some NPS are now included in MDT, their structures are continuously being altered by producers to avoid detection.^19^

The market availability of specific NPS is strictly connected to countries' respective legislation in place at the time of production and/or consumption.^20^, ^21^ This results in a constantly evolving market of NPS which presents the main analytical challenge for in‐field instruments and laboratories in charge to detect and quantify these substances. The large number of structurally diverse NPS available (~950 registered by UNODC^22^ and >4200 on the web^23^), and the pace at which these appear on the market (one new NPS per week^24^) are also contributing factors challenging detection, due to a lag in certified reference standard (RS) availability.^2^, ^3^ Low concentrations of potent NPS, for example, SCs or opioids, combined with inhomogeneous distribution on new matrices or formulations, employed to facilitate smuggling in prisons, are also factors making difficult their detection and identification.^25^

Currently, there are no universal globally agreed standard operating procedures (SOP) in place to identify TdA as well as NPS in prison. Drugs of abuse are often confiscated in this setting via cell, inmate, or visitor searches performed by prison officers.^26^ In some countries, such as the United Kingdom, the United States, and Canada, the use of sniffer dogs has also been reported for detection of TdA^26^ as well as SCs.^25^, ^27^ However, due to the ever‐changing nature of the NPS market, it is difficult to maintain the long‐term effectiveness of sniffer dogs with these substances.^9^ Once samples suspected to contain drugs are identified, these are screened using in‐field analytical techniques such as ion mobility spectrometry (IMS)^28^ and/or sent to external forensic laboratories for confirmatory analysis. External forensic laboratories employ traditional analytical techniques such as gas chromatography–mass spectrometry (GC–MS), liquid chromatography‐mass spectrometry (LC–MS), and nuclear magnetic resonance (NMR), which are costly and time consuming,^29^ but can give meaningful information even in the absence of RS. In addition, drug use can be identified by analysis of prisoners' biological specimens,^26^ which are also commonly sent to external forensic laboratories for analysis.

The aim of this manuscript is to investigate the current state of chemical detection and identification of NPS in prisons based on the available literature, looking at (I) the most predominant groups and specific NPS which have been reported in prison; (II) the routes and forms through which these were smuggled into prison, and (III) the analytical methods employed to detect and identify NPS in biological and non‐biological samples from prisons. A particular focus will be given to the UK situation for points (I) and (II). Recommendations are then presented in the future works section based on the findings of this review. To the best of the authors' knowledge, this marks the first systematic literature review examining detection in the prison setting for this complex and emerging group of substances.

## 2 METHODOLOGY

**FIGURE 1:**
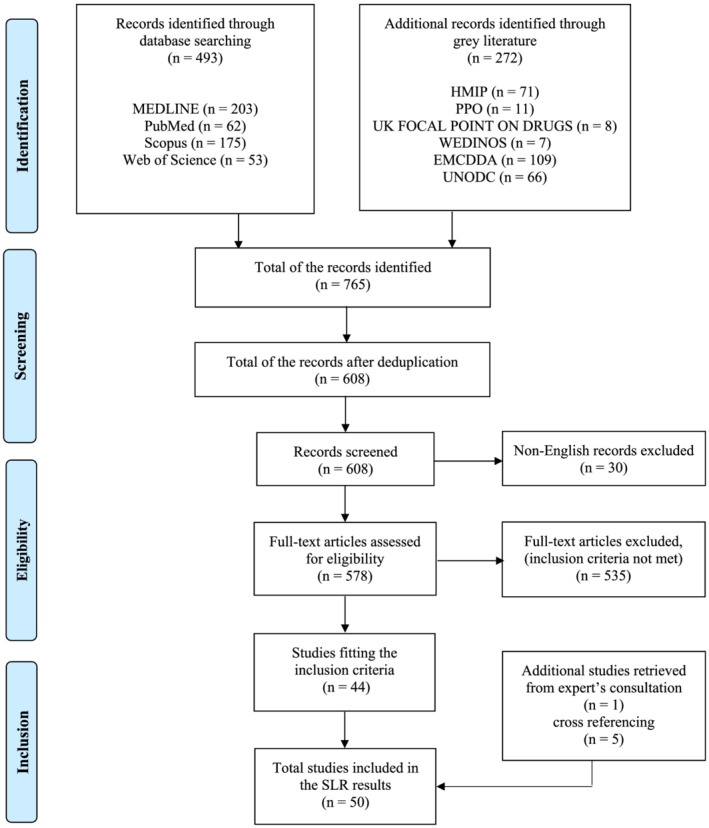
PRISMA flow diagram [Colour figure can be viewed at wileyonlinelibrary.com]

The methodology has been developed in line with the Preferred Reporting Items for Systematic reviews and Meta‐Analyses literature search extension (PRISMA‐S),^30^ which is a checklist employed to ensure that each component of a systematic literature search is completely reported, hence reproducible. Search words belonging to group 1 (including keywords such as NPS, NPS classes, and their synonyms) were combined using Boolean operators (OR/AND) to search words belonging to group 2 (including keywords such as prison and its synonyms) to give a search string, listed in full in the supporting information. The search was carried out between May 2020 and December 2021 using MEDLINE (EBSCO), Scopus (ELSEVIER), PubMed (NCBI), and Web Of Science (Clarivate) databases. A total of 493 citations were added to the review from the string search strategy. No study registries were searched. The grey literature search was carried out between May 2020 and December 2021 and included targeted hand‐searching of additional websites. A particular focus was on UK government and/or research organization websites, while also European and global agencies websites were consulted (supporting information). A total of 272 additional citations were added to the review from the grey literature search. The selected articles related to the topic were manually cross‐referenced to identify additional studies. A total of 2708 additional citations were added to the review from the cross‐referencing search. Some organizations were also contacted to enquire about the latest reports and/or additional unpublished data (e.g., UK focal point on drugs, Welsh Emerging Drugs & Identification of Novel Substances Project [WEDINOS], Office for National Statistics UK, and EMCDDA). No additional information sources or search methods were used. The search was not limited to any time or geographical restrictions. All languages were included in the search results; however, non‐English results were excluded during the review process. All document types available were searched on the databases; however, opinion/discussion papers, press release/magazines/websites articles, published conference abstracts, leaflets, posters, theses, protocols, and patents were excluded. No published filters were used in database searches, while some filters were used for the grey literature search (see supporting information). The comprehensive literature search on Scopus was finalized on December 2021; alerts were set up to provide updates of the literature in the form of weekly e‐mails, until the end of April 2021. While the other three databases were added at a later date and for consistency, the time limit was set to April 2021. The duplicates were removed using Microsoft Excel (Version 16.0.13426.20274) function to find and remove duplicates. The general methodology is outlined in the PRISMA flow diagram (Figure 1), while the details are reported in the supporting information. The methodology was independently checked/peer‐reviewed by the co‐authors Amira Guirguis and Jacqueline L. Stair.

## 3 RESULTS AND DISCUSSION

A total of 50 articles were identified via the systematic review process. Despite the global search, articles have been found to come only from a limited number of countries. Results, which can be divided amongst the three key themes, are presented and discussed below. Specific aspects of the literature may be presented in different sections for comparison purposes, to present key information according to each theme.

### 3.1 An overview of NPS reported in prisons

In order to have effective detection approaches, the NPS prisons' scene should be evaluated. This section provides an overview of NPS reported in the prison setting to date, based on the available resources. A variety of sources were identified including quantitative/qualitative self‐reporting studies, analytical studies of biological/non‐biological samples, organizational reports, and generic publications on NPS in prison. These were used to establish the NPS groups, as well as the specific compounds reported in prisons. Although some of the studies may be different in nature, the information on the NPS group or the specific name was collated to highlight overall observed trends in the literature. The number of publications related to NPS found in prison settings from 1978–2020 has increased from approximately 2010 to the present (Figure S1). No articles were found prior to 1978, and those found after 2010 mostly refer to the newly reported psychoactive substance phenomena. Most of the articles were quantitative/qualitative self‐reporting studies or generic publications on the topic; however, since 2017, interest in the chemical analysis of NPS seized in prisons has increased. For example, approximately 18% (*n* = 2) of the articles published in 2019 had an analytical perspective which increased to almost 38% (*n* = 6) in 2020. Overall, the increasing number of publications demonstrates the growing interest in this topic.

**FIGURE 2:**
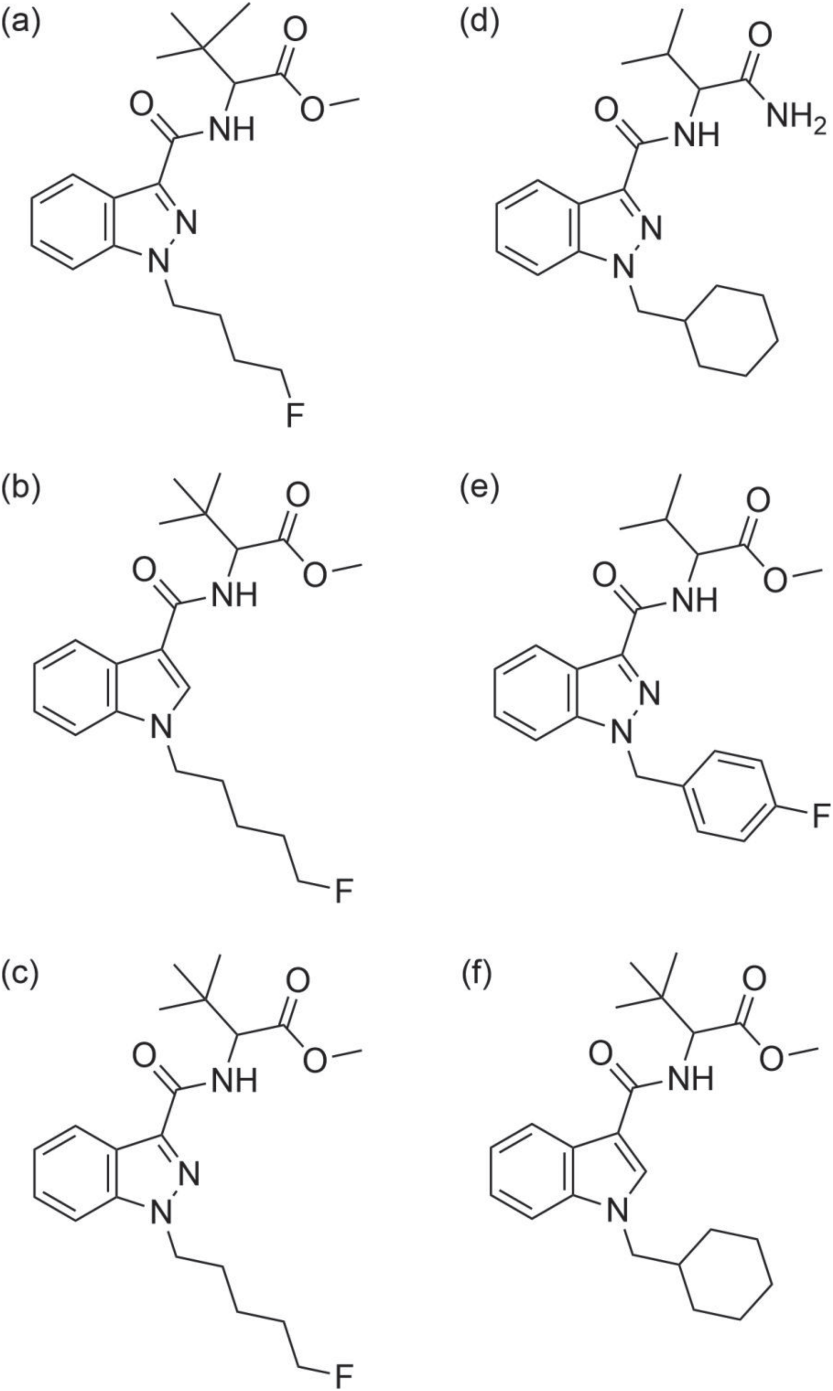
Chemical structures of the most reported NPS: (a) 4F‐MDMB‐BINACA, (b) 5F‐MDMB‐PICA, (c) 5F‐ADB, (d) AB‐CHMINACA, (e) AMB‐FUBINACA, and (f) MDMB‐CHMICA

An overview of the types of NPS reported in prisons is shown in Table 1. The NPS substance groups, reported in order of prevalence, were SCs, synthetic cathinones, synthetic opioids, benzodiazepines, stimulants, piperazines, and plant‐based substances. The predominant group of NPS that have been reported in prison were SCs, with a total of 63 different SCs and/or their metabolites. Due to the large number of SCs reported, this specific group was further divided into the nine relevant subgroups of alkoylindoles, benzoylindoles, carbazoles, γ‐carbolines, indole carboxylates, indole carboxamides, indazole carboxamides, naphthoylindoles, and 7‐azaindole carboxamides. The carboxamides were further divided into adamantly, cumylamine, valinamide, valinate, tert‐leucinamide, and tert‐valinate derived groups (Table 1). The most frequently referenced SCs were 4F‐MDMB‐BINACA (aka 4F‐MDMB‐BUTINACA),^21^, ^32^, ^34^, ^36^, ^38^, ^39^, ^40^, ^47^ 5F‐MDMB‐PICA,^5^, ^9^, ^19^, ^31^, ^33^, ^37^, ^38^, ^42^, ^43^, ^47^, ^48^, ^63^, ^64^, ^70^ 5F‐ADB (aka 5F‐MDMB‐PINACA),^4^, ^5^, ^21^, ^28^, ^33^, ^34^, ^38^, ^39^, ^45^ AB‐CHMINACA,^4^, ^19^, ^21^, ^28^, ^31^, ^42^ AMB‐FUBINACA (aka FUB‐AMB, MMB‐FUBINACA),^4^, ^5^, ^21^, ^25^, ^28^, ^38^ and MDMB‐CHMICA^21^, ^27^, ^31^, ^33^, ^42^, ^43^ (Figure 2). These all belong to the indazole and indole carboxamide subgroups of tert‐leucinamide, tert‐leucinate, valinamide, and valinate derived. The majority of studies were reported by Germany, the United Kingdom, and the United States. The availability of specific SCs is connected to the legislation related to their production, import, and export countries.^20^ For instance, when the People's Republic of China in 2018 placed under control 32 NPS, including 5F‐ADB, ADB‐FUBINACA, and AMB‐FUBINACA, a reduction in findings of these substances was registered across different countries.^21^, ^38^ A year later new SCs, structurally related to the latter but not covered by the legislative control, for example, MDMB‐4en‐PINACA and 4F‐MDMB‐BINACA, made their appearance on the market.^21^, ^38^, ^40^, ^41^, ^47^, ^48^ This highlights the evolving nature of the SC market, where trends are also reflected in the prison drug market.^21^, ^38^ Another example of the evolving SC market is related to the current loss of popularity of 5F‐PB‐22, which emerged and peaked from 2013 to 2015 in the United States^70^ and English prisons.^31^ The disappearance of 5F‐PB‐22 from the market was again due to its placement under control by the People's Republic of China in October 2015. Table 1 also identifies less common yet more recent SCs. In January 2019 in Germany, the γ‐carbolines derived 5F‐cumyl‐PEGACLONE was found for the first time in the post‐mortem blood and urine of a prisoner.^32^ The same SC was later found also in urine from German prisons along with cumyl‐CBMEGACLONE and cumyl‐PEGACLONE, also belonging to the γ‐carbolines.^21^ Other newly emerging SCs belonging to the subgroup 7‐azaindole carboxamides were 5F‐MDMB‐P7AICA and cumyl‐4CN‐B7AICA, also detected for the first time by urinalysis in German prisons in 2021.^21^

**TABLE 1:** NPS reported in prison identified via the systematic literature review

NPS group^a^, subgroup^b^ and name	Country	Reference
**SYNTHETIC CANNABINOIDS**		
Alkoylindoles		
5F‐UR‐144	England	^31^
FUB 144 (aka FUB‐UR‐144)	Germany	^21^
UR‐144	England	^31^
**Benzoylindoles**		
AM‐694	England	^31^
**Carbazoles**		
EG‐018	Germany	^21^
**γ‐Carbolines**		
5F‐cumyl‐PEGACLONE	Germany	^21^, ^32^
Cumyl‐CBMEGACLONE	Germany	^21^
Cumyl‐PeGaClone	Germany	^21^, ^28^
**Indole Carboxylates**		
5F‐PB‐22	England, Wales	^21^, ^31^, ^33^
PB‐22 (aka QUPIC)	England, Germany, Scotland	^28^, ^31^, ^34^
QUCHIC (aka BB‐22)	England	^31^
**Indole Carboxamides**		
5F‐MPP‐PICA	Scotland	^21^, ^34^
FUB‐PB‐22 (aka QUFUBIC)	England	^31^
**(a) Adamantly derived**		
STS‐135 (aka 5F‐APICA)	England	^31^, ^35^
**(b) Cumylamine derived**		
5F‐cumyl‐PICA	Germany	^21^
Cumyl‐CBMICA	Germany	^21^
**(c) Valinate derived**		
5F‐EMB‐PICA (aka EMB‐2201)	Scotland	^21^, ^34^, ^36^, ^37^
AMB‐4en‐PICA (aka MMB‐4 en‐PICA)	Germany	^21^
AMB‐FUBICA	Germany	^21^
MMB‐2201	Germany	^28^
MMB‐CHMICA (aka AMB‐CHMICA)	England, Scotland, USA	^5^, ^21^, ^25^, ^31^, ^34^, ^38^
**(d) Tert‐leucinamide derived**		
5F‐ABICA^c^	Germany	^21^
**(e) Tert‐leucinate derived**		
4F‐MDMB‐BICA	Belgium, Cyprus, France, Hungary, Lithuania, Slovenia, UK	^21^, ^34^, ^36^, ^37^
5F‐MDMB‐PICA^c^	Germany, UK, USA	^5^, ^9^, ^21^, ^34^, ^36^, ^37^, ^38^, ^39^, ^40^, ^41^
(*R*)‐5F‐MDMB‐PICA	Scotland	^20^
MDMB‐CHMICA	England, Germany, Wales	^21^, ^27^, ^31^, ^33^, ^42^, ^43^
**Indazole Carboxamides**		
THJ‐2201	England	^31^
**(a) Adamantly derived**		
5F‐APINACA (aka 5F‐AKB‐48)	England, Wales	^21^, ^27^, ^31^, ^33^, ^35^
APINACA (aka AKB‐48)	England, Germany	^21^, ^28^, ^31^, ^35^
FUB‐APINACA	Germany	^21^
**(b) Cumylamine derived**		
5F‐cumyl‐PINACA	England	^31^
Cumyl‐4CN‐BINACA (aka Cumyl‐CYBINACA)	Germany, Lithuania, UK, USA	^5^, ^21^, ^44^
Cumyl‐CBMINACA	Germany	^21^
**(c) Valinamide derived**		
AB‐CHMINACA	Germany, Lithuania, UK, USA	4, 19, 21, 28, 31, 42
AMB‐FUBINACA (aka FUB‐AMB, MMB‐FUBINACA)	England, Germany, Scotland, Wales, USA	^4^, ^5^, ^21^, ^25^, ^28^, ^38^
**(d) Valinate derived**		
5F‐AMB (aka 5F‐MMB‐PINACA, 5F‐AMB‐PINACA)	England, USA	^4^, ^31^
**(e) Tert‐leucinamide derived**		
5F‐AB‐PINACA	England, Germany	^21^, ^31^
5F‐ADB (aka 5F‐MDMB‐PINACA)	Germany, UK, USA	^4^, ^5^, ^21^, ^28^, ^33^, ^34^, ^38^, ^39^, ^45^
(*R*)‐5F‐ADB (aka (*R*)‐5F‐MDMB‐PINACA)	Scotland	^20^
5F‐ADB‐PINACA	England, Germany	^21^, ^31^
AB‐FUBINACA^c^	England, Germany	^21^, ^27^, ^31^, ^42^
ADB‐BINACA	Germany	^21^
ADB‐CHMINACA	Germany	^21^, ^42^
ADB‐FUBINACA	Germany, USA	^21^, ^42^, ^46^
**(f) Tert‐leucinate derived**		
4F‐MDMB‐BINACA (aka 4F‐MDMB‐BUTINACA)^c^	Germany, Scotland, Wales	^21^, ^32^, ^34^, ^36^, ^38^, ^39^, ^40^, ^47^
(*R*)‐4F‐MDMB‐BINACA	Scotland	^20^
MDMB‐4en‐PINACA^c^	Belgium, Cyprus, France, Germany, Hungary, Lithuania, Slovenia, UK, USA	^34^, ^36^, ^37^, ^38^, ^48^
(*R*)‐MDMB‐4en‐PINACA	Scotland	^20^
MDMB‐ChmINACA	Germany	^21^
MDMB‐FUBINACA (aka FUB‐MDMB, MDMB‐Bz‐F)	USA	^4^
**Naphthoylindoles**		
AM‐2201	Germany, England, Norway	^31^, ^42^, ^49^
JWH‐018	Norway	^21^, ^49^
JWH‐081	Germany	^21^
JWH‐122	Germany	^21^
JWH‐210	Germany	^21^
MAM‐2201	England	^31^
**7‐Azaindole Carboxamides**		
5F‐MDMB‐P7AICA	Germany	^21^
Cumyl‐4CN‐B7AICA	Germany	^21^
Non‐Specific (e.g., Spice)	Croatia, Cyprus, Czech Republic, Finland, France, Germany, Hungary, Ireland, Italy, Latvia, Lithuania, Norway, Poland, Slovenia, Sweden, and UK	^6^, ^8^, ^9^, ^10^, ^11^, ^17^, ^50^, ^51^, ^52^, ^53^, ^54^, ^55^, ^56^, ^57^, ^58^, ^59^, ^60^, ^61^, ^62^
**SYNTHETIC CATHINONES**		
4F‐PHP	Scotland	^38^
4‐MEC	England	^31^
Mephedrone	Australia, England	^31^, ^63^, ^64^
Methylone	Australia	^63^
Non‐Specific	Cyprus, Czech Republic, Finland, France, Germany, Hungary, Latvia, Lithuania, Poland, Sweden	^9^
**OPIOIDS**		
Acryloylfentanyl	Latvia	^65^
Carfentanil	Latvia	^66^
Cyclopropylfentanyl	Latvia	^67^
Non‐Specific	Czech Republic, Finland, Italy, Latvia, Poland, Sweden	^9^
**STIMULANTS**		
4‐methylmethamphetamine	England	^31^
Ethylphenidate	England	^27^, ^31^
Methylhexaneamine	England	^27^, ^31^
Methiopropamine	England	^27^, ^31^
**BENZODIAZEPINES**		
Etizolam	England	^27^
Non‐Specific	Finland, Italy, Latvia, Poland	^9^
**PIPERAZINES**		
1‐benzylpiperazine	Sweden	^68^
**PLANT‐BASED**		
Dihydrokavain	England	^31^
**PHENCYCLIDINE‐TYPE**		
Methoxphenidine	England	^27^

^a^ The NPS groups were adapted from UNODC Word Drug Report 2020.^16^

^b^ The NPS subgroups were adapted from Abate et al.^69^

^c^ Includes the NPS and its metabolites.

SCs were also often reported in general studies but referred to by their street name “Spice”; these studies are collated under the “non‐specific” subgroup in Table 1. A high level of self‐reported use of SCs in England was documented by the “Spice awareness project” (unpublished work)^8^ undertaken in a category C prison in November 2014, by a charity. It reported that 80% of prisoners tried “Spice” during their current sentence, while around 65% admitted to using “Spice” currently.^8^ Similar results were found in a report published in May 2016, which surveyed 684 prisoners across nine English prisons. It found that 33% of prisoners reported having used “Spice” in the last month, making it the most popular misused substance amongst hooch (illegally brewed alcohol), cannabis, heroin substitutes, and heroin. Interestingly, around 66% of survey respondents thought that more than half of the prisoners in their prison used “Spice.” In contrast, a survey by Her Majesty Inspectorate of Prisons (HMPI) distributed to inmates in eight prisons between June and November 2014 (*n* = 1376) reported that only 10% had used “Spice” during their current sentence.^8^ The discrepancies in the results of the self‐reported studies might be confounded by the differing level of trust prisoners exhibit towards the organizations conducting the studies.^6^

Synthetic cathinones, synthetic opioids, new benzodiazepines, and stimulants were also found. In 2017, 10 European countries reported synthetic cathinones being used in their prisons according to the EMCDDA.^9^ Additionally, two subsequent studies reported the detection of mephedrone^31^, ^63^, ^64^ and 4‐methylethcathinone (4‐MEC)^31^ in English prisons. Synthetic cathinones were also reported in an Australian wastewater analysis (WWA) study, which identified methylone and mephedrone in a small prison facility.^63^ Synthetic opioids were less reported in European prisons in comparison with SCs and cathinones. Studies reporting opioid usage were confined mainly to the North‐Eastern area of Europe and in Italy.^9^ Specifically, a total of 10 seizures of synthetic opioids were reported from prisons in Latvia, including acryloylfentanyl, carfentanil, and cyclopropylfentanyl.^65^, ^66^, ^67^ In England, etizolam was identified sprayed onto letters that were seized and analysed in 2015,^27^ where up to three SCs, stimulant NPS such as ethylphenidate, methoxphenidine, methiopropramine, and adulterants were also detected. It is not well understood why low potency NPS and adulterants were found in conjunction with SCs in this matrix, and it was not possible to ascertain whether these were intentionally added to achieve enhanced desirable effects^27^ or perhaps to hinder identification.

To a lesser extent, substances belonging to the NPS groups of piperazines, plant‐based, and phencyclidine‐type have also been reported in prison settings. For example, the NPS 1‐benzylpiperazine was detected in Sweden between 2000 and 2002 in 11 post‐mortem biological samples.^68^ In some of the analysed specimens, traces of amphetamines were also found. It was unclear whether the prisoners intended to take the piperazine analogue or believed that it was amphetamine. Kava (*Piper methysticum)* is a plant that grows in the South Pacific Islands with both stimulant and depressant effects on the central nervous system (CNS); its kavalactone, dihydrokavain, was found in three pre‐release and one voluntary drug testing (VDT) urine specimens in a prison near Manchester (UK) in 2015.^31^ A thematic report by HMPI suggests that despite some differences, drug use in prison reflects, to some extent, the use in the general population.^8^ Generally, in both prison and the general population, drugs with a depressant effect on the CNS are preferred over their stimulant counterparts.^71^ Yet, higher use of stimulants in the general population is suggested by Bonds and Hudson (2015) where the results of urinalysis showed a 3.5‐fold increase in stimulant detection for prisoner admission (“on reception” samples) versus incarcerated residents (“pre‐release,” MDT and VDT samples).^31^ Despite the latter study took place in 2015, it is the only study available reporting data on different NPS classes, including stimulants, in the prison setting. The results from Table 1, including literature/reports outside of the United Kingdom, also follow an NPS prison trend favouring SCs, used to relieve stress and boredom of imprisonment.^6^, ^8^, ^10^

### 3.2 NPS smuggling routes and forms

Knowledge of potential smuggling routes and forms, which may differ from that of TdA,^9^, ^72^ could help inform and support the prison security system in tackling detection and identification of NPS. A total of 26 studies related to NPS smuggling routes and forms, employed to illegally introduce NPS in prisons, were retrieved from the literature.

Seventeen out of 26 studies applied to the NPS smuggling routes employed in prisons and were divided into seven groups (Figure S2). The postal service was highlighted as the most prevalent smuggling route (*n* = 14) for bringing NPS into prisons through parcels or mail.^8^, ^9^, ^10^, ^19^, ^25^, ^27^, ^33^, ^45^, ^46^, ^49^, ^64^, ^68^, ^73^ Due to the trend of spraying NPS on paper, some UK prisons photocopied prisoners' correspondence, which reduced smuggling but was time‐consuming. However, in some circumstances (e.g., English and Welsh prison “Rule 39”^74^ and Scotland “Legal Mail”^75^) legal, confidential correspondence can only be opened and inspected by prison staff in specific situations, which makes photocopying and routine checks more complicated.^76^ The second most reported NPS smuggling routes, each described in five articles, were concealment inside the body and transportation over prison walls. Concealment in body orifices,^6^, ^9^, ^10^, ^46^, ^60^ for example, gastrointestinal system, rectum, or vagina, is particularly challenging to detect^72^; new prisoners were found to smuggle up to 280 g of SCs through this route.^10^ X‐ray body scanners able to detect drugs concealed inside the body or under clothes are being introduced in more UK prisons to tackle the issue. Transportation over prison walls was also reported via drones^6^, ^8^, ^9^, ^10^, ^49^ or using catapults.^8^ NPS thrown over the wall were found to be concealed also in unusual items, such as carcasses of birds^6^, ^9^ or oranges.^9^ To overcome this issue, some prisons installed nets around the perimeters or used radar systems to intercept drones. Lesser reported NPS smuggling routes included via new prisoners or prisoners who were released on bail,^6^, ^9^, ^10^ prison staff,^6^, ^8^, ^10^ visitors,^8^, ^9^, ^10^ and external contractors^9^ including cleaning companies, waste disposal trucks, and canteen distributors.

Twenty‐five studies applied to the forms in which NPS are smuggled into prison. The forms reported for NPS in prison (Figure S3) were via paper matrices, herbal mixtures, food and drinks, solid materials, clothes, cosmetics, and e‐liquids. Paper matrices (*n* = 19), commonly delivered by postal services or during social visits, were the main form reported that was used to smuggle NPS^6^, ^9^, ^10^, ^12^, ^47^, ^59^, ^60^, ^61^, ^77^ as confirmed by analytical studies performed on seized samples.^5^, ^20^, ^21^, ^25^, ^27^, ^28^, ^34^, ^38^, ^41^ The term “paper matrices” is used to encompass letters, children's drawings, blank paper sheets, greeting cards, photographs, books, documents, poems, blotters, paper snippets, Bible pages, online printed catalogues, rice paper, crosswords, and sudoku puzzles.^5^, ^6^, ^9^, ^10^, ^12^, ^20^, ^21^, ^25^, ^27^, ^28^, ^34^, ^38^, ^41^, ^47^, ^59^, ^60^, ^61^, ^77^, ^78^ Prisoners are believed to take NPS, specifically SCs, by licking, chewing, swallowing, smoking,^27^ or placing in eyes^21^ the paper, which is usually cut into 1 cm^2^ or smaller pieces.^28^ When in this formulation and size, such samples are easily concealed, carried, and traded between inmates.^38^ SCs are commonly produced in solid form, then dissolved in an organic solvent such as acetone, and easily sprayed onto paper matrices^10^ or herbal material.^31^ Recently, other general reports also highlight paper matrices as the most popular form to smuggle NPS in prison across Europe, especially in Finland, Germany, Hungary, Lithuania, Poland, and Sweden^5^, ^9^ because of the challenges in detection.^8^ The second most prevalent form reported was herbal mixtures^21^, ^28^, ^31^, ^38^, ^45^, ^64^, ^66^, ^77^ (*n* = 8). In particular, herbal/plant material such as marshmallow (*Althea officinalis*) leaves^31^ or tobacco^28^, ^64^ were mixed or sprayed with SCs. In UK prisons, inmates were found smoking cigarettes laced with SCs infused herbs,^38^ yet after the smoking ban was implemented (2018), NPS were found infused in paper inserted between the heating constituent and the cartridge of e‐cigarettes.^21^ The increased risk of fatal and non‐fatal overdoses, related to the consumption of SCs in the forms discussed, could also be due to their heterogeneous distribution on the matrices.^41^ Areas with a high drug concentration on paper are known as “hot‐spots,”^9^ while those on herbal mixtures are known as “hot‐pockets.”^79^ Moreover, this has implications for chemical detection, making representative sampling by the analyst challenging.^31^, ^38^ Apirakkan et al. determined analytically the presence of SCs dissolved in vaping liquid,^25^ purchased from internet retailers before the 2016 UK “legal high ban.” In 2021, SCs in vaping liquid were also found in Welsh prisons, accounting for only 0.6% of SC samples analysed^21^; however, the popularity of this new formulation could grow due to detection difficulty and will require monitoring in the future. NPS were also found in the lid of soft drinks^10^ and in the form of pre‐sealed food packages such as crackers, coffee, and instant noodles.^9^ SCs were also seized in solid, powder, and crystalline forms^21^, ^31^, ^67^ as well as found sprayed on clothing^60^ and textiles^28^ in prison settings. Lastly, acryloylfentanyl, a new synthetic opioid, was detected in a cosmetic cream in a Latvian prison.^65^

### 3.3 NPS detected in non‐biological and biological prison samples

The studies in which NPS were detected in non‐biological and biological samples from prisons are summarized in Tables 2 and 3, respectively. SCs were the most reported group of NPS in both samples matrices.^4^, ^5^, ^20^, ^21^, ^31^, ^32^, ^34^, ^35^, ^38^, ^40^, ^41^, ^43^, ^46^, ^48^, ^49^ Based on the results of our review, 5F‐APINACA (aka 5F‐AKB‐48) (337 findings), 4F‐MDMB‐BINACA (aka 4F‐MDMB‐BUTINACA) (273 findings), 5F‐PB‐22 (273 findings), MDMB‐4en‐PINACA (246 findings), 5F‐MDMB‐PICA (141 findings), and 5F‐ADB (aka 5F‐MDMB‐PINACA) (131 findings) were the most reported NPS in seizures. While the most detected SCs in biological samples were 5F‐AKB‐48 (1449 findings), MDMB‐CHMICA (584 findings), 5F‐MDMB‐PICA (388 findings), 4F‐MDMB‐BINACA (301 findings), MDMB‐4en‐PINACA (166 findings), and AB‐FUBINACA (124 findings). The above specific SCs results are skewed by the extensive number of samples analysed in the study carried out by Bonds and Hudson in English prisons, where 39% of seized samples (*n* = 1088) and 17.9%/16.9% of phase I (*n* = 7395)/phase II (*n* = 1833) urine samples tested positive for SCs.^31^ Although this study gives an indication to the extent SCs are used in prisons it is dated back to 2015 and does not necessarily reflect current prison trends on specific SCs. A more recent study by Norman et al. (2021) reported SCs found in prison seizures between 2018 and 2020 in Scotland and Wales.^21^ In this study, the most prevalent were 4F‐MDMB‐BINACA (aka 4F‐MDMB‐BUTINACA) (244 findings) MDMB‐4en‐PINACA (209 findings), and 5F‐ADB (aka 5F‐MDMB‐PINACA) (179 findings). The same study also reported a 33.6% incidence of SC detection in urine samples from German prisons^21^ of which 5F‐MDMB‐PICA (376 findings), 4F‐MDMB‐BINACA (aka 4F‐MDMB‐BUTINACA) (297 findings), and MDMB‐4en‐PINACA (165 findings) were the most reported. This study, carried out internationally, showed that some similarities between countries such as Germany, England, Wales, and the United States, were present, for example, high prevalence of 5F‐MDMB‐PINACA, which are usually driven by legislation in countries producing NPS and international control. However, differences are seen as well; for instance, γ‐carbolinone SCs were often found in Germany, yet rarely seen in UK and US prisons.

**TABLE 2:** Summary of NPS detected in non‐biological prison samples

NPS detected	Sample form	Analytical technique	Method	Country	Sample's year	Reference
AMB‐FUBINACA and MMB‐CHMICA	Paper	UHPLC‐MS/Q‐Orbitrap^a^	Qualitative	England	N.A.	^25^
5F‐AKB‐48, AB‐FUBINACA, ethylphenidate, etizolam, MDMB‐CHMICA, methiopropamine, methylphenidate and methoxphenidine	Paper	UPLC‐MS/QToF^b^	Qualitative	England	2016	^27^
(*S*) and (*R*)‐4F‐MDMB‐BINACA, (*S*) and (*R*)‐5F‐MDMB‐PICA, (*S*) and (*R*)‐5F‐MDMB‐PINACA, (*S*) and (*R*)‐MDMB‐4en‐PINACA	Paper	Chiral HPLC‐MS/QToF	Qualitative	Scotland	2018–2020	^20^
4F‐PHP, 4F‐MDMB‐BINACA, 5F‐MDMB‐PICA, 5F‐MDMB‐PINACA, AMB‐CHMICA, AMB‐FUBINACA and MDMB‐4en‐PINACA	Paper	GC‐MS^c^; UPLC‐MS/QToF and NMR^d^	Quantitative	Scotland	2018–2019	^38^
4F‐MDMB‐BINACA and 5F‐MDMB‐PICA	Paper	GC‐MS and HPLC‐MS/QtoF	Qualitative	Germany	2019	^41^
4F‐MDMB‐BUTINACA, 4F‐MDMB‐BUTINACA 2′‐indazole isomer, 5F‐ADB and 5F‐MDMB‐PICA	Paper	GC‐MS	Qualitative	USA	2019	^5^
4F‐MDMB‐BICA, 4F‐MDMB‐BINACA, 5F‐EMB‐PICA, 5F‐MDMB‐PICA, 5F‐MPP‐PICA, 5F‐MDMB‐PINACA, AMB‐CHMICA, MDMB‐4en‐PINACA and PB‐22.	Paper	IMS^e^; GC‐MS and UPLC‐MS/QtoF	Qualitative	Scotland	2018–2020	^34^
4F‐MDMB‐BICA, 4F‐MDMB‐BINACA, 5F‐EMB‐PICA, 5F‐MDMB‐PICA, 5F‐MDMB‐PINACA, 5F‐MPP‐PICA and MDMB‐4en‐PINACA	Paper	GC‐MS and UPLC‐MS/QtoF	Qualitative	Scotland	2018–2020	^21^
4F‐MDMB‐BINACA, 5F‐APINACA, 5F‐MDMB‐PINACA, 5F‐PB‐22, AMB‐FUBINACA, MDMB‐4en‐PINACA and MDMB‐CHMICA	Herbal mixture, solid, paper, e‐liquid	UPLC‐MS/QtoF	Qualitative	Wales		
5F‐ADB (5F‐MDMB‐PINACA), AB‐CHMINACA, APINACA, cumyl‐PEGaClone, FUB‐AMB, MMB‐2201 and PB‐22	Herbal mixture, paper	IMS and GC‐MS	Qualitative	Germany	N.A.	^28^
5F‐AKB‐48, 5F‐AMB, 5F‐PB‐22, 5F‐UR‐144, AB‐CHMINACA, AB‐FUBINACA, AKB‐48, AM‐2201, FUB PB‐22, MAM‐2201, MDMB‐CHMICA, PB‐22, QUCHIC, STS‐135 and UR‐144	Herbal mixtures	GC‐MS	Qualitative	England	2014–2015	^31^
5F‐AKB‐48, 5F‐PB‐22, AB‐FUBINACA, AKB‐48, AM‐2201, mephedrone, PB‐22 and STS‐135	Herbal mixtures	UPLC‐MS/QtoF	Qualitative	England	2014–2015	^64^

^a^ Ultra‐high performance liquid chromatography‐mass spectrometry/quadrupole‐orbitrap.

^b^ Ultra‐performance liquid chromatography‐mass spectrometry/quadrupole time of flight.

^c^ Gas chromatography‐mass spectrometry.

^d^ Nuclear magnetic resonance.

^e^ Ion mobility spectrometry.

**TABLE 3:** Summary of NPS detected in biological prison samples

NPS detected	Sample Form	Analytical technique	Method	Country	Sample's year	Reference
4‐MEC, 4‐methylmethamphetamine, 5F‐AB‐PINACA, 5F‐ADB‐PINACA, 5F‐AKB‐48, 5F‐PB‐22, 5F‐UR‐144, AB‐CHMINACA, AB‐FUBINACA, ADB‐FUBINACA, AKB‐48, AM‐2201, AM‐694, cumyl‐5F‐PINACA, ethylphenidate, FAM‐2201, dihydrokavain, mephedrone, methiopropamine, methylhexaneamine. MAM‐220, MDMB‐CHMICA, STS‐135, THJ‐018, THJ‐2201 and UR‐144	Urine	UHPLC‐MS/LTQ‐Orbitrap^a^ and UHPLC‐MS/Q‐Orbitrap	Qualitative	England	2014‐2015	^31^
SCRAs	Urine	Immunoassay				
3rd generation adamantly SCRA	Urine	UPLC‐MS/QToF	Qualitative	England	N.A.	^35^
4F‐MDMB‐BICA, 4F‐MDMB‐BINACA, 5F‐ABICA amide hydrolysis metabolite, 5F‐AB‐PINACA, 5F‐ADB‐PINACA, 5F‐cumyl‐PEGACLONE, 5F‐cumyl‐PICA, 5F‐MDMB‐P7AICA, 5F‐MDMB‐PICA, 5F‐MDMB‐PINACA, AB‐FUBINACA amide hydrolysis metabolite, AB‐CHMINACA, ADB‐BINACA, ADB‐CHMINACA, ADB‐FUBINACA, AMB‐4en‐PICA, AMB‐CHMICA, AMB‐FUBICA, cumyl‐4CN‐B7AICA, cumyl‐4CN‐BINACA, cumyl‐CBMEGACLONE, cumyl‐CBMICA, cumyl‐CBMINACA, cumyl‐PEGACLONE, EG‐018, FUB‐144, FUB‐APINACA, JWH‐081, JWH‐122, JWH‐210, MDMB‐4en‐PINACA, MDMB‐CHMINACA	Urine	UHPLC‐MS/TQ^b^	Qualitative	Germany	2018‐2020	^21^
4F‐MDMB‐BINACA 3,3‐dimethylbutanoic acid and 5F‐MDMB‐PICA 3,3‐dimethylbutanoic acid	Urine	UHPLC‐MS/QToF	Qualitative	USA	2019	
1‐benzylpiperazine	Urine	GC‐MS	Qualitative	Sweden	2000‐2002	^68^
5F‐ADB^c^, 5F‐AMB^c^, AB‐CHMINACA^c^, FUB‐AMB^c^ and MDMB‐FUBINACA^c^	Blood, urine	UPLC‐MS/TQ	Quantitative	USA	2017‐201	^4^
5F‐cumyl‐PEGACLONE and 5F‐cumyl‐PEGACLONE^d^	Blood, urine	UHPLC‐QLIT^e^	Quantitative	Germany	2019	^32^
ADB‐FUBINACA	Blood, urine	UHPLC‐MS/QToF	Quantitative	USA	N.A.	^46^
MDMB‐4en‐PINACA 3,3‐dimethylbutanoic acid	Blood	UHPLC‐MS/QToF	Qualitative	USA	2019	^48^
4F‐MDMB‐BINACA and 5F‐MDMB‐PICA	Blood	GC‐MS and HPLC‐MS/QToF	Quantitative	Germany	N.A.	^40^
MDMB‐CHMICA	Blood	UPLC‐MS/QToF	Qualitative	England	N.A.	^43^
AM‐2201 and JWH‐018	Saliva	UPLC‐MS/TQ	Qualitative	Norway	N.A.	^49^
Mephedrone and methylone	Wastewater	UHPLC‐MS/QLIT	Quantitative	Australia	2013	^63^

^a^ Ultra‐high performance liquid chromatography‐mass spectrometry/linear trap quadrupole‐orbitrap.

^b^ Ultra‐performance liquid chromatography®‐mass spectrometry/triple quadrupole.

^c^ Identified by their butanoic acid conjugated metabolites.

^d^ Metabolites.

^e^ Ultra‐high performance liquid chromatography‐mass spectrometry/quadrupole linear ion trap.

#### 3.3.1 Analysis and sample preparation of NPS in non‐biological matrices

Seized NPS were mainly found impregnated into paper or herbal material in the prison setting (Table 2). Literature findings revealed that the mainstay analytical techniques employed for the analysis of non‐biological prison samples were liquid chromatography (LC)^20^, ^21^, ^25^, ^27^, ^34^, ^38^, ^41^, ^64^ or gas chromatography (GC)^5^, ^21^, ^28^, ^31^, ^34^, ^38^, ^41^ coupled with mass spectrometry (MS), which are regarded as highly discriminatory techniques for forensic analysis of drugs.^73^ Table 2 shows a prevalence for the use of high‐resolution mass spectrometry (HRMS) such as QToF^20^, ^21^, ^27^, ^34^, ^38^, ^41^, ^64^ and quadrupole‐orbitrap.^25^ In seven studies, GC–MS was employed either as stand‐alone^5^, ^31^ or alongside other techniques.^21^, ^28^, ^34^, ^38^, ^41^ The in‐field technique IMS was evaluated in two studies screening for SCs in prison.^28^, ^34^

Bonds and Hudson developed an analytical workflow for general seized material in prison.^31^ First, unknown powders were analysed by colorimetric tests while tablets were compared against the TICTAC database, then in some cases further analysed either by IR or GC–MS. Herbal matrices were analysed by GC–MS. Only 15 different SCs in the form of herbal material were detected, and no other NPS were found in these prison seizures.^31^ Herbal matrices were reported in an additional three studies where extraction was performed using pure methanol^21^, ^28^, ^64^ or ethanol.^31^ Some studies reported centrifugation and withdrawal of supernatant^64^ or filtration of the extracts^28^ to reduce impurities introduced in the chromatographic column. Ford and Berg (2016) were able to detect a wide range of substances with different polarities in seized herbs using UPLC–MS/QToF with two simultaneous screening methods,^64^ a “NOIDS screen” (>100 SCs) and “general screen” (>1300 drugs and metabolites). It was more common to see samples containing only one SC^21^, ^28^, ^31^, ^64^; however, some samples contained multiple SCs. Up to eight SCs were found in one samples by Bonds and Hudson.^31^ The majority of studies found in Table 2 characterized NPS on paper seized in prison. Ford and Berg were the first to present analytical evidence of NPS smuggled on paper. In this study, as well as Apirakkan et al., sniffer dogs were used initially to detect SCs on paper which were then sent for further analysis.^25^, ^27^ In general, paper matrices were sampled using areas ranging from 0.70 to 1 cm^2^ ^5^, ^20^, ^27^, ^28^, ^38^, ^41^; for example, a biopsy punch was employed by Norman et al. to ensure sampling consistency. Samples were extracted for 5 to 20 min^25^, ^27^, ^38^, ^41^ either in pure methanol^5^, ^20^, ^25^, ^27^, ^28^, ^38^, ^41^ or a combination of methanol and dichloromethane (25:75),^38^ in order to cover compounds with different polarities. To extract substances from paper, one extraction was used in most studies.^5^, ^20^, ^25^, ^27^, ^28^, ^38^, ^41^ McKenzie and co‐workers^38^ spiked paper with known quantities of SCs (i.e., 5F‐MDMB‐PICA, 4F‐MDMB‐BINACA, 5F‐MDMB‐PINACA, AMB‐FUBINACA, and AMB‐CHMICA) and showed that 94–98% was recovered after one extraction in 25:75 methanol/dichloromethane with approximately 100% recovery when three consecutive extractions were performed. In the case of Hascimi et al., a paper sample was seized from a deceased inmate's cell who tested positive for 4F‐MDMB‐BINACA metabolite in urine. Analysis of the paper sample showed no SCs using GC–MS; however, further analysis with the more sensitive HPLC‐MS/QtoF identified 4F‐MDMB‐BINACA and 5F‐MDMB‐PICA.^41^ This case highlights the importance of determining typical concentrations for NPS and specifically SCs on paper to determine which methods/techniques are the most suitable for these sample types. To this end, McKenzie and co‐workers used a combination of two techniques for identification and quantification of SCs infused in paper seized in Scottish prisons between 2018 and 2019.^38^ This was the first report on SC concentrations in paper samples (*n* = 145). The SCs quantified by GC–MS, collected by 3× extraction, were the following: 5F‐MDMB‐PICA (*n* = 59, <0.08 ± 0.01 to 0.76 ± 0.11 mg/cm^2^ paper); 4F‐MDMB‐BINACA (*n* = 45, <0.09 ± 0.01 to 0.94 ± 0.14 mg/cm^2^ paper); 5F‐ADB (*n* = 42, <0.05 ± 0.01 to 1.17 ± 0.17 mg/cm^2^ paper); MDMB‐4en‐PINACA (*n* = 22, <0.07 ± 0.01 to 0.58 ± 0.09 mg/cm^2^ paper); AMB‐FUBINACA (*n* = 5, 0.20 ± 0.03 to 1.16 ± 0.17 mg/cm^2^ paper); and AMB‐CHMICA (*n* = 1, 0.58 ± 0.09 mg/cm^2^ paper).^38^ Furthermore, concentration mapping showed a variability of 5‐fold for AMB‐CHMICA across seized paper ranging between 0.47 and 2.38 mg/cm^2^ ^38^ demonstrating the inhomogeneity of SCs across paper samples linked to the drying process employed. A study by Caterino et al. evaluated the impact of latent fingerprint detection (i.e., exposure to 1,8‐diazaflouren‐9‐one [DFO] and ninhydrin) on the extraction and detection of SC impregnated paper.^5^ The presence of four SCs, 4F‐MDMB‐BUTINACA, 5F‐ADB, 5F‐MDMB‐PICA, and the 2′‐indazole isomer of 4F‐MDMB‐BUTINACA, were successfully identified by GC–MS before and after fingerprint analysis, as well as in the ninhydrin run‐off. Although SCs were detected in all three scenarios, quantitative analysis would be helpful to assess the concentration reductions encountered due to this type of processing. In an effort to distinguish SC optical isomers found on paper, Antonides et al. used two chiral columns (i.e., a Phenomenex Lux® Amylose‐1 and Lux® i‐Cellulose‐5 [5 μm, 4.6 × 100 mm]) coupled to a HPLC‐photo diode array (PDA)‐MS/QToF method to analyse 177 SC infused paper samples seized in Scottish prisons between 2018 and 2020.^20^ SCs were the enantiopure (*S*)‐enantiomer in >89% of the samples, although in 2–16%, the (*R*)‐enantiomer was detected as well. This study highlighted the potential for chiral profiling of chiral valinate and *tert*‐leucinate based SCs to distinguish production batches of drugs for intelligence purposes.

The in‐field technique IMS was evaluated in two studies screening for SCs in prison.^28^, ^34^ Generally, laboratory‐based hyphenated techniques are regarded as confirmatory techniques, while in‐field techniques are employed as a preliminary test. Quick, minimal, and non‐destructive sample preparation makes IMS well‐suited for in‐field analysis by non‐expert users; for example, the analytes were collected by rubbing a Teflon sample trap on the sample's surface.^28^, ^34^ Metternich et al. evaluated both simulated and prison casework samples using the IMS IONSCAN600®.^28^ The simulated samples contained mixtures of 5F‐ADB and five TdA/prescription only medicine (POM) (100 ng for each compound) concealed in cosmetic and food samples, while 36 casework samples were mainly in herbal and paper form. The IMS identified 5F‐ADB in most of the matrices evaluated (i.e., 9 of 11), but failed in highly viscous matrices (e.g., toothpaste or liquid soap). For the casework samples, 12 samples (mainly herbal material) tested positive for SCs which were confirmed by GC–MS analysis. In contrast, Norman et al. focused on the detection of SCs in seized paper samples (*n* = 392) to evaluate the operational reliability of two Ion Trap Mobility Spectrometers (ITMS®), the Rapiscan Itemiser® 3E and 4DN.^34^ Sampling was performed on paper of varying sizes which resulted in high trap loading variability.^34^ A limited but tailored IMS library, composed of nine “Spice alarm,” was employed to detect the SCs using the reduced mobility (K_0_)^28^ and drift time.^34^ The study found that the level of agreement between ITMS® and GC–MS results was 91.1% for the Itemiser® 3E and 92.9% for the Itemiser® 4DN instruments. Reasons for disagreement included both false negative (e.g., no IMS alarm generated for trace or multiple SCs present) and false positive (e.g., Spice, buprenorphine, and cocaine alarms generated by IMS, but not detected by GC–MS) results. The Itemiser® 3E was more suitable for the detection of SCs due to its ability to detect cumyl compounds (cumyl‐4CN‐BINACA and 5F‐cumyl‐PEGACLONE), compared with the Itemiser® 4DN. The observed LODs (oLOD) were determined for nine SCs and ranged from 0.5 to 100 ng and 5 to 500 ng for the Itemiser® 3E and Itemiser® 4DN (Region 0) instruments, respectively. It was highlighted that variability of the K_0_ values for the compounds between different instruments could lead to misidentification or false negatives.^34^ Reduced selectivity can occur as substances that exhibit a difference <0.025 cm^2^ V^−1^ s^−1^ in their K_0_ values cannot be discriminated unambiguously,^80^ for example, 5F‐PB‐22 and AB‐CHMINACA with K_0_ values of 0.9995 and 0.9975 cm^2^ V^−1^ s^−1^, respectively.^28^ On the other hand, newly emerging SCs with structural similarity to the compounds already in the library can potentially be identified,^34^ based on the overlapping K_0_ values.^80^ Nonetheless, IMS has difficulty when detecting more than one analyte in a mixture, where only the analyte with higher peak intensity is detected by the instrument. For example, in a sample containing a mixture of AB‐CHMINACA, APINACA, 5F‐ADB, MMB‐2201, and caffeine, only 5F‐ADB was detected.^28^ Additionally, the low LOD, in the ng range,^28^, ^34^ could lead to false positives due to cross‐contamination, arising from papers collected and stored in the same evidence bag.^34^

#### 3.3.2 Analysis and sample preparation of NPS in biological matrices

In this section, articles including post‐mortem analysis of specimen,^4^, ^32^, ^40^, ^48^ case studies of prisoners admitted to hospital following NPS intake,^35^, ^43^, ^46^ as well urine,^21^, ^31^, ^68^ saliva^49^ or wastewater^63^ analysis carried out on prison samples are presented (Table 3). In general, biological samples were pretreated, before NPS extraction, by the addition of buffers (i.e., acetate,^31^ phosphate,^21^, ^31^, ^40^ or carbonate^21^, ^32^, ^35^) and/or pH manipulation by addition of sodium hydroxide^68^ or trisaminomethane (TRIS) HCl^4^, ^48^ to reduce enzymatic activity and preserve the NPS. The main analytical technique employed for the analysis of biological samples (i.e., 12 out of 13 studies) was liquid chromatography coupled with mass spectrometry (LC–MS) or tandem mass spectrometry (MS/MS). Different MS analysers or their combination such as triple quadrupole,^4^, ^21^, ^49^ quadrupole‐time of flight,^21^, ^35^, ^40^, ^43^, ^46^, ^48^ linear trap quadrupole‐orbitrap,^31^ and quadrupole‐linear ion trap^63^ were employed. These analysers were all equipped with HRMS‐MS capabilities except for the triple quadrupole. Additionally, the use of GC–MS^40^, ^68^ and one immunoassay^31^ were also reported.

Urine was the biological matrix most reported for antemortem detection of NPS consumed by prisoners.^21^, ^31^, ^35^, ^46^, ^68^ The preference for this matrix can be explained by the low invasiveness of the collection technique, and the longer detection window of drug metabolites (days‐weeks), when compared with blood matrices (hours‐day). However, urine is susceptible to factors including quantity collected, pH, difference in individual metabolism which may influence the quantitative results.^81^ Additionally, urinalysis leads to minimal parent drug detection, while being more useful for the identification of metabolites. For instance, different SCs can undergo different metabolic reactions in the human liver and form the same metabolite, thus making the identification of the exact SC ambiguous, for example, 5F‐ADBICA amide hydrolysis metabolite may result from 5F‐ABICA, 5F‐AMB‐PICA, or 5F‐EMB‐PICA metabolism.^21^ However, in a clinical rather than a forensic context, this is not always disadvantageous as demonstrated by Rook et al., which employed the metabolite 1‐adamantylamine as a urine marker to quickly identify adamantly‐type SCs, for example, 5F‐AKB‐48, AKB‐48, and STS‐135 in an emergency context.^35^ Extraction of the NPS from urine samples was performed by standard techniques such as precipitation, filtration, liquid–liquid extraction (LLE),^31^, ^35^, ^68^ and solid‐phase extraction (SPE).^21^, ^31^, ^40^ Bonds and Hudson employed a reversed‐phase SPE (i.e., Agilent Nexus polymer sorbent) and extracted analytes with different polarities such as non‐SC NPS, OTC/POM, and TdA.^31^ Similarly, a SPE cartridge based on a bimodal non‐polar and strong cation exchange (SCX) mechanism (i.e., Agilent Bond Elut Certify cartridge) was also effective at extracting SCs along with other drugs (e.g., cocaine and amphetamine‐like substances).^40^ Lastly, Norman et al. employed a non‐polar and anion exchange SPE cartridge (i.e., Agilent Bond Elut Plexa PAX) effective for SCs and their metabolites.^21^ In an effort to recover NPS for quantification purposes, β‐glucuronidase enzymes^4^, ^21^, ^31^ were often added to urine samples to hydrolyse glucuronide metabolites back to the parent drug. Rook et al. employed the UPLC‐MS/QToF qualitative methods previously described by Ford and Berg^64^ for the analysis of non‐biological samples. Similar to Ford and Berg,^64^ Bonds and Hudson employed simultaneously two screening methods with a UHPLC–MS/LTQ‐Orbitrap and a UHPLC–MS/Q‐Orbitrap to detect NPS in urine specimens (i.e., a “general screen” and a “SCRA screen,” respectively) to cover a wider range of substances with different polarities, increasing the chance of positive detection.^31^ The UHPLC–MS/TQ system, employed for the analysis of urine samples from German prisons, successfully identified, on full scan, 31 SCs and metabolites, which were then confirmed in multiple reaction monitoring (MRM) mode.^21^ Several studies also utilized a stable isotopically labelled (SIL) IS to correct for analyte loss during sample preparation,^4^, ^31^, ^68^ for example, hydroxypentyl JWH‐018‐d5. SCs in biological matrices are not usually detected through GC–MS methods^4^ due to low concentration and the requirement of a derivatization step before analysis. In one case, GC–MS was successfully employed to analyse urine samples (i.e., derivatization via fluorinated anhydride) from 11 prisoners for the emerging NPS, 1‐benzylpiperazine.^68^ A point of care (POC) test was also trialled for the screening of urine samples from prisons. The “Spice” immunoassay “dip and read” was externally validated on urine samples (*n* = 514); it gave a positive SC match for only 1.4% of the samples tested versus 20% confirmed by UPLC–MS/MS.^31^ A high number of results (*n* = 96) were likely false negatives, while 0.2% false positives were recorded. This highlighted limitations in coverage and sensitivity; therefore, the authors did not recommend the use of such immunoassay.^31^ When performing immunoassays, the usefulness of false positives, which may be due to the cross‐reactivity of substances presents in a sample that have similar characteristics, must be noted.

Blood samples were used in post‐mortem^4^, ^32^, ^40^ or antemortem analysis in hospitalized and unresponsive prisoners^43^, ^46^ as it involves a more invasive collection by trained staff. In general blood specimens are more challenging to handle and store due to putrefaction and autolysis processes^81^ especially when post‐mortem. Blood analysis enables mainly detection of the parent drug in contrast to urine analysis^81^; however, it is possible to detect the metabolite in blood as well, for example, MDMB‐4en‐PINACA 3,3‐dimethylbutanoic acid detected in post mortem femoral blood.^48^ The characteristic and quality (i.e., pH level, presence of clots, and water quantity) of blood specimen is strictly related to the site of blood collection, for example, central, or peripheral. Central blood, due to post‐mortem redistribution, contains increased drug levels,^32^ which may compromise exact quantification; hence, analysis of femoral blood is preferable. For example, Giorgetti et al. found a higher quantity of the novel SC 5F‐cumyl‐PEGACLONE in central (0.22 ng/ml) versus femoral (0.12 ng/ml) blood.^32^ The addition of SIL IS, for example, JWH‐200‐d5 in this case, was employed for accurate quantification purposes of SCs. This study also highlighted the challenges with the lack of data on post‐mortem redistribution and toxic concentration ranges in the assessment of the toxicological significance score of SCs. In contrast, higher concentrations (34–17 ng/ml) of the SC ADB‐FUBINACA were detected in the serum of a “body packer” after the containment was compromised.^46^ To target low SC concentrations, Kleis et al.^40^ reported a LC‐MS/QToF qualitative screening approach run in auto‐MS/MS, a data‐independent acquisition (DIA) scan mode in conjunction with a preferred SC list. This was used to identify 5F‐MDMB‐PICA and 4F‐MDMB‐BINACA in the femoral blood of an inmate, which were then quantified and found to be 0.14 and 0.48 ng/ml, respectively. In addition, Krotulski et al. also used a DIA scan mode termed MS/MS^ALL^ with SWATH® acquisition which records the MS/MS of every molecule in the sample which led to the detection of MDMB‐4en‐PINACA metabolite in a forensic toxicological case of an inmate.^48^ A data mining approach which is the retrospective analysis of data files acquired under non‐targeted conditions to determine the presence of drugs that were not tested for at the time of first data processing, was also applied to the samples analysed by these authors. Meyyappan et al. employed the same UPLC‐MS/QToF qualitative methods previously described by Ford and Berg^64^ and Rook et al.^35^

Lastly, NPS were also detected in saliva and wastewater. As the need for easy and non‐invasive collection of biological specimens is increasing Øiestad et al.^49^ validated a screening method for SCs using a commercially available oral fluid collection device. Time to sampling was highlighted as a key factor for the analysis of this matrix, due to the high enzymatic activity in the saliva. However, stability issues were overcome by the addition of a preservative solution in the vial of the collection device made of chlorhexidine digluconate, Tween® 20, Flag Blue dye and deionized water, followed by storage at 4°C. During the analysis a large ion enhancement up to 6000% was recorded, due to the use of the preservative solution. A diazepam‐d5 IS was added during sample preparation, yet was not useful as it eluted earlier in the run, highlighting the importance of accurate selection of IS. This method also offered the advantage of detecting the parent drugs instead of the metabolite; however, the potential for adulteration or contamination should be considered. Wastewater analysis (WWA) was carried out in a small Australian prison to assess drug use and to compare its result to urinalyses.^63^ This approach allowed a daily representation of drugs used by prisoners; for instance, on day 12, 537 mg (3–5 daily doses) of methylone were detected. Mephedrone was also detected but concentrations were below the quantification limit (<0.0001–<0.025 μg/L) of the UHPLC–MS/quadrupole linear ion trap (QLIT) employed. When WWA and urinalyses were compared, no methylone was detected by urinalyses due to the different type of sampling. This highlights the advantage of WWA in gaining a daily picture of the overall use of drugs in contrast to routine urinalyses, which are often targeted. However, it was unfeasible to discern between the prisoner and staff/visitor's contribution.^63^

## 4 CONCLUSIONS

This study reviewed the NPS reported in prisons, ways and forms in which they are smuggled, and analytical methods used to detect them. SCs were by far the dominant NPS group reported, followed to a lesser extent by synthetic cathinones, synthetic opioids, new benzodiazepines, and stimulants. Specifically, SCs belonging to the last generation subclasses of the tert‐leucinate indazole carboxamides (i.e., 4F‐MDMB‐BINACA and MDMB‐4en‐PINACA), tert‐leucinate indole carboxamides (i.e., 5F‐MDMB‐PICA), and tert‐leucinamide indazole carboxamides (i.e., 5F‐ADB) were the most reported in recent findings. The literature suggests that most NPS, in particular SCs, are smuggled via paper and herbal matrices into prison, predominantly using postal services. For paper samples, one solvent extraction was sufficient for identification via chromatography‐mass spectrometry (i.e., LC‐HRMS/MS and GC–MS), while SC quantitative studies reported concentrations between 0.05–1.17 mg/cm^2^ providing parameters for further development of in‐field methods. In particular, in‐field monitoring by sniffer dogs and IMS were able to detect SCs on paper and shows promise for rapid NPS detection on this matrix. However, IMS suffers from reduced selectivity where substances cannot be discriminated unambiguously.^80^ Laboratory‐based technique, chromatography‐mass spectrometry, was most often employed for the analysis of NPS in biological samples (i.e., LC‐HRMS/MS) from prison. Whilst detection of the exact NPS in a forensic context is important to gather intelligence; in a clinical/emergency context of decision making, identification of metabolites as being quicker can be more useful. The application of sample mining and data mining approaches to seized and urine samples can help gain a bigger picture of emerging NPS and their metabolites and to determine when a substance first appeared. The authors would like to highlight the following limitations of the study: (I) a particular focus was given to the UK grey literature (e.g., Her Majesty Prison and Probation Services and Prison and Probation Ombudsman reports) and (II) it was not possible to determine NPS trends in prisons overtime due to a lack of detail reported in the available literature (e.g., different seizure years or missing years).

## 5 FUTURE WORK

Based on the outcomes of this review, specific areas are suggested for future work. As SCs were smuggled principally via paper and herbal matrices, rapid and accurate in‐field analysis of these sample forms would improve real‐time decision‐making. Due to the evolving market, focus should be given to monitoring effectiveness of current in‐field techniques for identifying new emerging SCs. For instance when IMS fails to identify SCs in suspected samples which produce peaks in the typical SC detection range, it should be used in conjunction with a laboratory‐based prison drugs monitoring program.^34^ As a result of the reduced selectivity and inability of IMS to detect more than one substance in a mixture, future research should also focus on other in‐field technologies. It should be noted that spectroscopic techniques such as Raman and FTIR, are powerful analytical techniques,^29^ that can discriminate between NPS in tablet and powder forms, and between NPS isomers. These are also non‐destructive and available in handheld technology; however, they struggle with interfering matrices especially if containing a low amount of NPS, such as herbal material,^31^ paper matrices or tobacco.^28^ The use of approaches such as surface enhanced Raman spectroscopy (SERS) using minimally invasive sampling methods could be investigated to promote practical application of SCs detection on paper and herbal matrices. Of particular interest is the application of SERS swabs and colloids, embedded with metal nanoparticles to enhance the Raman signal, already employed for the screening of TdA and NPS.^82^ A methcathinone spectrum was obtained in the study performed by Lee et al. where 23 μg of the analyte was deposited into SERS active films made of hydroxyethylcellulose polymer and aggregated silver nanoparticles. The samples were wiped with a cotton bud wetted then pressed onto a pre‐swelled SERS substrate. Conveniently, the film when dry is similar to paper and can be stored for a year and cut to size when needed.^83^ While Yu et al. designed paper‐based inkjet‐printed SERS swabs able to collect trace amounts of analyte from large surface areas, which can be concentrated into a small‐volume SERS‐active region by lateral‐flow concentration. The swabs were validated for the detection of 5 μg of heroin and 5 μg of cocaine on glass slides. The measurements show that the technique is quantitative and repeatable across multiple swabs.^84^ The easy sampling approach similar to IMS could allow rapid yet selective identification of NPS in herbal and paper matrices.

As immunoassays lacked accuracy, there is still a need to develop sensitive, real‐time and non‐invasive POC testing to screen for SCs in biological samples (i.e., urine and oral fluids) for use in a decision‐making context during on‐site intoxication and emergencies. The IMS (IONSCAN LS®) with a high pressure injection system^85^ was proven effective for detecting TdA gamma‐hydroxybutyrate (GHB) and gamma hydroxyvalerate (GHV) in synthetic urine at approximately 3 μg/ml, which suggests the method could potentially work for saliva samples. More recently the same instrument was employed for the detection of cocaine in saliva.^86^ However, their field collection device, based on a cotton swab with an indicator and a molecularly imprinted polymer (MIP) sorbent, was designed to selectively retain cocaine. Therefore, adaptation of such device to retain SCs would be needed. Moreover, fluorescence spectral fingerprinting combined with numerical modelling could be used to identify the likely presence of SCs, as well as provide more specific information on structural class and concentration (∼1 μg/ml). This approach can detect both parent and combusted material, and it is practical for detecting SCs in oral fluids.^87^ All the procedures mentioned in the above studies^85^, ^86^, ^87^ could be employed by non‐specialized personnel. For the development of new laboratory‐based LC–MS detection methods for detection of NPS in biological samples, HRMS incorporating DIA should be preferred, as this will allow the application of sample mining and data mining. While to monitor NPS general trends in prisons and for intelligence purposes, WWA analysis would provide a more representative picture of the overall extent of substance use, compared with MDT. WWA is already used for TdA in Australia and trialled in the United States and Spain.^26^ This approach compared with MDT is more cost‐effective and less invasive. Recently an air monitoring approach, already employed for the detection of NPS^88^ was evaluated for detection of SCs. Paul et al.^89^ employed a combination of fixed and mobile sampling units, worn by prison officers, coupled with thermal desorption (TD) sorbent tubes, allowing for multiple location sampling. A two‐dimensional gas chromatography (GCxGC)‐MS/ToF method was validated for AB‐FUBINACA, UR144, MDMB‐4en‐PINACA, and MDMB‐CHMCA; however, these SCs were not found in the collected samples. Therefore further investigation on wide applicability of the technique to detect SCs in prisons should be considered.

## Supporting information


**Table S1**. Key words related to the systematic literature review
**Table S2**. Synthetic cannabinoids reported in non‐biological samples by country and year
**Table S3**. Synthetic cannabinoids reported in biological samples by country and year
**Table S4**. Common, street and IUPAC names of NPS mentioned in the review
**Figure S1**. Trends of scientific publications of NPS reported in prison settings from 1978–2020
**Figure S2**. Routes in which NPS are smuggled into prison
**Figure S3**. Forms in which NPS are smuggled into prison
