# Supplementary material for: NPS detection in prison: A systematic literature review of use, drug form, and analytical approaches
Source: Drug Test Anal. 2022 Apr 20;14(8):1350–67. doi: 10.1002/dta.3263 (PMC9545023; doi:10.1002/dta.3263)
Supplement: Supplementary file 1 — Table S1. Key words related to the systematic literature review Table S2. Synthetic cannabinoids reported in non‐biological samples by country and year Table S3. Synthetic cannabinoids reported in biological samples by country and year Table S4. Common, street and IUPAC names of NPS mentioned in the review Figure S1. Trends of scientific publications of NPS reported in prison settings from 1978–2020 Figure S2. Routes in which NPS are smuggled into prison Figure S3. Forms in which NPS are smuggled into prison [file DTA-14-1350-s001.docx]

**Supporting Information- Full methodology strategy**

**Database search**

The databases search (KEY WORDS- TITLE -ABSTRACT) was conducted on Scopus (<http://www.scopus.com>) between May 2020 and January 2021. While MEDLINE (<https://www.ebsco.com/products/research-databases/medline-complete>), PubMed (https://pubmed.ncbi.nlm.nih.gov/) Web of Science (<https://access.clarivate.com/login?app=wos&alternative=true&shibShireURL=https:%2F%2Fwww.webofknowledge.com%2F%3Fauth%3DShibboleth&shibReturnURL=https:%2F%2Fwww.webofknowledge.com%2F%3Fmode%3DNextgen%26action%3Dtransfer%26path%3D%252Fwos%252Fwoscc%252Fbasic-search%26DestApp%3DUA&referrer=mode%3DNextgen%26path%3D%252Fwos%252Fwoscc%252Fbasic-search%26DestApp%3DUA%26action%3Dtransfer&roaming=true>) were searched between August 2021 and December 2021. The search was performed using the two-word groups (Table 1) to give a search string. To note that for the databases Scopus and PubMed the string was split in three parts as only a limited number of digits in the search bar were allowed. Boolean operators 1) OR was used to combine words within groups while 2) AND was used to combine words between groups. Double quotation was used to search for a two or more words near each other. While the wildcard * represented any number of characters, e.g., prison* is equal to prison, prisons, prisoner and prisoners.

Table 1. Key words related to the systematic literature review

| **Word group 1** | **Word group 2** |
| --- | --- |
| "psychoactive substance" | prison* |
| "legal high" | jail |
| "designer drug" | penitentiary |
| NPS | “correctional service” |
| "synthetic cannabinoid" | “correctional institution” |
| SCRA |  |
| "NPS opi*" |  |
| "NPS benzodiazepine" |  |
| "phencyclidine-type" |  |
| "ketamine-type" |  |
| "plant-based NPS" |  |
| aminoindane |  |
| tryptamine |  |
| piperazine |  |
| phenethylamine |  |
| "synthetic cathinone" |  |
| Notes: Boolean operators 1) OR combined with words within groups 2) AND combined between groups. Double quotation is used to search for a phrase, while the wildcard * represents any number of characters, even zero. | |

The full search strings strategy performed on **MEDLINE**, lead to n= **203** results (updated on the 31/04/21) and is detailed below:

One unique string: "NPS" OR "synthetic cannabinoid" OR "SCRA" OR "designer drug" OR "psychoactive substance" OR "legal high" OR "NPS opi*" OR "NPS benzodiazepine" OR "Phencyclidine-type" OR "Plant-based NPS" OR "Aminoindane" OR "Tryptamine" OR "Piperazine" OR "Phenethylamine" OR "Synthetic cathinone" AND prison* OR jail OR penitentiary OR "correctional services" OR "correctional institution" (203 results).

The full search strings strategy performed on **PubMed**, lead to n= **62** results and is described below:

1A-part 1) "NPS" OR "synthetic cannabinoid" OR "SCRA" OR "designer drug" OR "psychoactive substance" OR "legal high" AND prison* OR jail OR penitentiary OR "correctional service" OR "correctional institution" (59 results)

1A-part 2) "NPS opi*" OR "NPS benzodiazepine" OR "phencyclidine-type" OR "plant-based NPS" OR "aminoindane" OR "tryptamine" OR "piperazine" OR "phenethylamine" AND prison* OR jail OR penitentiary OR "correctional service" OR "correctional institution" (3 results)

1A-part 3) "synthetic cathinone" AND prison* OR jail OR penitentiary OR "correctional services" OR "correctional institution" (0 results)

The full search strings strategy performed on **Scopus**, lead to n= **175** results (updated on the 31/04/21 through alerts) and is described below:

1A-part 1) "NPS" OR "synthetic cannabinoid" OR "SCRA" OR "designer drug" OR "psychoactive substance" OR "legal high" AND prison* OR jail OR penitentiary OR "correctional service" OR "correctional institution" (162 results)

1A-part 2) "NPS opi*" OR "NPS benzodiazepine" OR "phencyclidine-type" OR "plant-based NPS" OR "aminoindane" OR "tryptamine" OR "piperazine" OR "phenethylamine" AND prison* OR jail OR penitentiary OR "correctional service" OR "correctional institution" (13 results)

1A-part 3) "synthetic cathinone" AND prison* OR jail OR penitentiary OR "correctional services" OR "correctional institution" (0 results)

The full search strings strategy performed on **Web of Science**, lead to n= **53** results and is described below:

One unique string: ("NPS" OR "synthetic cannabinoid" OR "SCRA" OR "designer drug" OR "psychoactive substance" OR "legal high" OR "NPS opi*" OR "NPS benzodiazepine" OR "Phencyclidine-type" OR "Plant-based NPS" OR "aminoindane" OR "Tryptamine" OR "Piperazine" OR "Phenethylamine" OR "Synthetic cathinone") AND (prison* OR jail OR penitentiary OR "correctional services" OR "correctional institution") (53 results).

The search ﻿was not associated with any time or geographical restrictions; all languages were included in the search results; however non-English results were removed during the review process. All the document type available were searched on the databases.

After the second update on the Scopus search done on the 31/01/21 the alert for each string was set up to provide updates of the literature in the form of weekly e-mails, until the end of April 2021. This led to an additional 6 articles, of which 1 met the SLR inclusion criteria and it was accepted. While the other databases were added at a later date, however, the time limit was set to April 2021 for consistency.

The full databases literature search led to a total n= **493** documents.

**Grey literature search**

The grey literature search was conducted between May 2020 and January 2021 (31/01/21) and included targeted hand searching of websites listed below. A particular focus was put on UK government and/or research organization websites (from 1 to 4), while also European (5) and global (6) agencies websites were consulted.

1. HM Inspectorate of Prisons (<https://www.justiceinspectorates.gov.uk/hmiprisons/> ), which was searched in the section “Our reports” for the following report type: “Annual report” and “Thematic reports and research” selecting only reports from 2010 to 2020 without any location restriction (“Annual report” 23/05/20 n=12; “Thematic reports and research” 10/06/20 n=59).
2. Prisons & Probations Ombudsman (<https://www.ppo.gov.uk> ), which was searched in the section “Reports and Publications” for the following report type: “Annual reports” and “Learning lesson reports” selecting only reports from 2010 to 2020 without any other restriction (“Annual report” 22/05/20 n=11).
3. UK Focal Point on Drugs (<https://www.gov.uk/government/publications/united-kingdom-drug-situation-focal-point-annual-report> ) which was searched in the section “Annual Reports” selecting only reports from 2010 to 2020 without any other restriction (10/05/20 n=8). Additionally, the organisation was e-mailed on date 18/05/20 to enquire about the reports for the years 2018 and 2019, which has not been yet published.
4. WEDINOS (<https://www.wedinos.org/>) which was searched in the section “Newsletter” selecting only annual reports from 2013 to 2020 (as before 2013 the service/organisation was not active hence no reports were produced) without any other restriction (04/06/20 n=7).
5. EMCDDA (<https://www.emcdda.europa.eu/>), which was searched in the section “Publications” categorised by “keyword(s)” for “NPS” (“NPS” 09/06/20 n=109)
6. UNODC (<https://www.unodc.org/>), which was searched in the section “What do we do?” then “Research” by selecting “Synthetic drugs” and then clicking on “SMART Publications” icon selecting only reports from 2010 to 2021 and excluding poster and leaflets as not an acceptable academic source (09/06/20 n=66)

The grey literature search led to an additional total n=**272** documents.

Also, EMCDDA newsletter for new publications on the topic was subscribed. While where setting an alert was not possible, the search was rerun the date prior the paper submission (31/01/21) to ensure everything was kept up to date.

Some of the grey literature searches ﻿were associated with some time restrictions (specified above for each organisation website/database) as from preliminary searches we found that prior the 2010 the use of NPS was not spread in the prison setting. Only research document, annual reports and thematic paper were searched on government and/or research organisation websites. Some organisations were also contacted to enquiry about the latest reports and/or additional unpublished data (e.g., UK FOCAL POINT ON DRUGS, WEDINOS, Office for National Statistics UK and EMCDDA). None of the above led to additional record.

**Inclusion/exclusion criteria**

inclusion criteria for this systematic literature review will be peer reviewed empirical research studies (quantitative/qualitative self-reporting studies; analytical studies of biological/non-biological samples; organisational reports and generic publications) and organisational reports and publications, which explore, evaluate or relate to 1) the use and 2) the forms and ways in which New Psychoactive Substances (NPS) are smuggled in prison setting. The search was limited to studies published in the English language.

Studies that considered the following were excluded:

- NPS use related to community settings (e.g., offenders on conditional release, detainees in police custody, any testing done on admission to prison).
- Opinion/discussion papers, letters to editor which were not peer reviewed, press release/magazines/websites articles, published conference abstracts, leaflets, posters, thesis, protocols and patents.
- Systematic Literature reviews, as categorised as a secondary source of information (however these were cross referenced to find primary pieces of information otherwise not accessible)
- Studies where due to the lack breakdown by specific class, it was not possible to extract data correctly.

**Study Selection**

The abstracts of identified papers were assessed against the inclusion and exclusion criteria by GV and AM. Discrepancies were resolved through meetings between GV, AM, JS and AG. Following this process, full papers were retrieved for review by GV or AM.

**Deduplication of results**

A total of n=765 of results were retrieved from the database search the grey literature and the Scopus alert. The duplicates were removed using the Excel function to find and remove duplicated. After deduplication the total number of results dropped to n= 608, as n=157 of duplicates was found.

**Additional studies**

A n=1 (Van Dyken et al.) additional study was found through expert’s advice/consultation.

**Cross referencing**

The reference list of the included articles (n=44) found through the search string, grey literature search and expert’s advice/consultation, was cross referenced. Additionally, also the articles added to the review from the cross-referencing search (n=6) were cross referenced. This process led to a total of n=1937 additional citations which were manually searched to identify additional pertinent studies.

Table 2. Synthetic cannabinoids reported in non-biological samples by country and year

| **Scotland** | **2018** | **2019** | **2020** | **Tot.** | **Reference** |
| --- | --- | --- | --- | --- | --- |
| Cumyl-4CN-BINACA | 1 | 0 | 0 | 1 | Norman et *al.*, 2021 |
| 4F-MDMB-BINACA | 0 | 94 | 61 | 155 | Norman et *al.*, 2021 |
| 5F-MDMB-PINACA | 22 | 23 | 3 | 48 | Norman et *al.*, 2021 |
| AMB-FUBINACA | 4 | 2 | 0 | 6 | Norman et *al.*, 2021 |
| EMB-FUBINACA | 2 | 2 | 0 | 4 | Norman et *al.*, 2021 |
| MDMB-4en-PINACA | 0 | 63 | 109 | 172 | Norman et *al.*, 2021 |
| 4F-MDMB-BICA | 0 | 0 | 8 | 8 | Norman et *al.*, 2021 |
| 5F-EMB-PICA | 0 | 0 | 11 | 11 | Norman et *al.*, 2021 |
| 5F-MDMB-PICA | 2 | 80 | 46 | 128 | Norman et *al.*, 2021 |
| 5F-MPP-PICA | 0 | 0 | 1 | 1 | Norman et *al.*, 2021 |
| AMB-CHMICA | 0 | 2 | 0 | 2 | Norman et *al.*, 2021 |
| **Wales** | **2018** | **2019** | **2020** | **Tot.** | **Reference** |
| 5F-PB-22 | 0 | 4 | 0 | 4 | Norman et *al.*, 2021 |
| 4F-MDMB-BINACA | 0 | 77 | 12 | 89 | Norman et *al.*, 2021 |
| 5F-APINACA | 0 | 4 | 0 | 4 | Norman et *al.*, 2021 |
| 5F-MDMB-PINACA | 28 | 103 | 0 | 131 | Norman et *al.*, 2021 |
| AMB-FUBINACA | 16 | 33 | 0 | 49 | Norman et *al.*, 2021 |
| MDMB-4en-PINACA | 0 | 26 | 11 | 37 | Norman et *al.*, 2021 |
| MDMB-CHMICA | 0 | 1 | 0 | 1 | Norman et *al.*, 2021 |
| **England** | **2015** |  |  | **Tot.** | **Reference** |
| 5F AKB-48 | 317 | No data | No data | 317 | Bond and Hudson, 2015 |
| 5F AMB | 12 | No data | No data | 12 | Bond and Hudson, 2015 |
| 5F PB-22 | 258 | No data | No data | 258 | Bond and Hudson, 2015 |
| 5F UR-144 | 7 | No data | No data | 7 | Bond and Hudson, 2015 |
| AB-CHMINACA | 11 | No data | No data | 11 | Bond and Hudson, 2015 |
| AB-FUBINACA | 11 | No data | No data | 11 | Bond and Hudson, 2015 |
| AKB-48 | 6 | No data | No data | 6 | Bond and Hudson, 2015 |
| AM-2201 | 16 | No data | No data | 16 | Bond and Hudson, 2015 |
| FUB-PB-22 | 1 | No data | No data | 1 | Bond and Hudson, 2015 |
| MAM-2201 | 4 | No data | No data | 4 | Bond and Hudson, 2015 |
| MDMB-CHMICA | 59 | No data | No data | 59 | Bond and Hudson, 2015 |
| PB-22 | 1 | No data | No data | 1 | Bond and Hudson, 2015 |
| QUICHIC | 69 | No data | No data | 69 | Bond and Hudson, 2015 |
| STS-135 | 3 | No data | No data | 3 | Bond and Hudson, 2015 |
| UR-144 | 1 | No data | No data | 1 | Bond and Hudson, 2015 |
| **England** | **2016** |  |  | **Tot.** | **Reference** |
| 5F-APINACA (aka **5F-AKB-48**) | 5 | No data | No data | 5 | Ford & Berg, 2018 |
| AB-FUBINACA | 3 | No data | No data | 3 | Ford & Berg, 2018 |
| MDMB-CHMICA | 4 | No data | No data | 4 | Ford & Berg, 2018 |
| **England** | **2014/2015** |  |  | **Tot.** | **Reference** |
| 5F-APINACA (aka 5F-AKB-48) | 10 | No data | No data | 10 | Ford & Berg, 2016 |
| 5F-PB-22 | 11 | No data | No data | 11 | Ford & Berg, 2016 |
| AB-FUBINACA | 2 | No data | No data | 2 | Ford & Berg, 2016 |
| AM-2201 | 1 | No data | No data | 1 | Ford & Berg, 2016 |
| APINACA (aka AKB-48) | 1 | No data | No data | 1 | Ford & Berg, 2016 |
| PB-22 (aka QUPIC) | 1 | No data | No data | 1 | Ford & Berg, 2016 |
| STS-135 (aka 5F-APICA) | 1 | No data | No data | 1 | Ford & Berg, 2016 |
| **England** | **N\A** |  |  | **Tot.** | **Reference** |
| AMB-FUBINACA (aka FUB-AMB) | 1 | No data | No data | 1 | Apikkaran et al. 2020 |
| MMB-CHMICA (aka AMB-CHMICA) | 1 | No data | No data | 1 | Apikkaran et al. 2020 |
| **Germany** | **N\A** |  |  | **Tot.** | **Reference** |
| 5F-ADB (aka 5F-MDMB-PINACA) | 6 | No data | No data | 6 | Metternich *et al.*, 2018 |
| AB-CHMINACA | 1 | No data | No data | 1 | Metternich *et al.*, 2018 |
| AMB-FUBINACA (aka FUB-AMB) | 5 | No data | No data | 5 | Metternich *et al.*, 2018 |
| APINACA (aka AKB-48) | 3 | No data | No data | 3 | Metternich *et al.*, 2018 |
| Cumyl‐PeGaClone | 3 | No data | No data | 3 | Metternich *et al.*, 2018 |
| MMB‐2201 | 1 | No data | No data | 1 | Metternich *et al.*, 2018 |
| PB-22 (aka QUPIC) | 1 | No data | No data | 1 | Metternich *et al.*, 2018 |
| **Germany** |  | **2019** |  | **Tot.** | **Reference** |
| 4F-MDMB-BINACA (aka 4F-MDMB-BUTINACA) | No data | 1 | No data | 1 | Hascimi et *al.*, 2020 |
| 5F-MDMB-PICA | No data | 1 | No data | 1 | Hascimi et *al.*, 2020 |
| **US** |  | **2019** |  | **Tot.** | **Reference** |
| 4F-MDMB-BINACA (aka 4F-MDMB-BUTINACA) | No data | 1 | No data | 1 | Caterino *et al.,* 2019 |
| 4F-MDMB-BINACA ﻿2'-indazole isomer | No data | 1 | No data | 1 | Caterino *et al.,* 2019 |
| 5F-ADB (aka 5F-MDMB-PINACA) | No data | 1 | No data | 1 | Caterino *et al.,* 2019 |
| 5F-MDMB-PICA | No data | 1 | No data | 1 | Caterino *et al.,* 2019 |

Table 3. Synthetic cannabinoids reported in biological samples by country and year

| **Germany** | **2018** | **2019** | **2020** | **Tot.** | **Reference** |
| --- | --- | --- | --- | --- | --- |
| JWH-081 | 0 | 1 | 1 | 2 | Norman *et al.*, 2021 |
| JWH-122 | 0 | 0 | 1 | 1 | Norman *et al.*, 2021 |
| FUB-144 | 0 | 0 | 1 | 1 | Norman *et al.*, 2021 |
| EG-018 | 0 | 1 | 0 | 1 | Norman *et al.*, 2021 |
| 5F-Cumyl-PEGACLONE | 28 | 32 | 1 | 61 | Norman *et al.*, 2021 |
| Cumyl-CBMEGACLONE | 0 | 0 | 4 | 4 | Norman *et al.*, 2021 |
| Cumyl-CHMEGALONE | 0 | 1 | 0 | 1 | Norman *et al.*, 2021 |
| Cumyl-PEGACLONE | 5 | 2 | 0 | 7 | Norman *et al.*, 2021 |
| Cumyl-4CN-BINACA | 1 | 0 | 0 | 1 | Norman *et al.*, 2021 |
| Cumyl-CBMINACA | 0 | 0 | 6 | 6 | Norman *et al.*, 2021 |
| 5F-Cumyl-PICA | 0 | 1 | 0 | 1 | Norman *et al.*, 2021 |
| Cumyl-CBMICA | 0 | 5 | 11 | 16 | Norman *et al.*, 2021 |
| Cumyl-4CN-B7AICA | 2 | 1 | 0 | 3 | Norman *et al.*, 2021 |
| 5F-MDMB-P7AICA | 4 | 4 | 2 | 10 | Norman *et al.*, 2021 |
| 4F-MDMB-BINACA | 0 | 200 | 97 | 297 | Norman *et al.*, 2021 |
| 5F-AB-PINACA | 10 | 0 | 4 | 14 | Norman *et al.*, 2021 |
| 5F-ADB-PINACA | 4 | 0 | 0 | 4 | Norman *et al.*, 2021 |
| 5F-MDMB-PINACA | 79 | 15 | 4 | 98 | Norman *et al.*, 2021 |
| ADB-BINACA | 0 | 0 | 22 | 22 | Norman *et al.*, 2021 |
| AB-CHMINACA | 3 | 0 | 1 | 4 | Norman *et al.*, 2021 |
| ADB-CHMINACA | 3 | 0 | 0 | 3 | Norman *et al.*, 2021 |
| MDMB-CHMINACA | 2 | 0 | 0 | 2 | Norman *et al.*, 2021 |
| FUB-APINACA | 3 | 0 | 0 | 3 | Norman *et al.*, 2021 |
| AB-FUBINACA amide hydrolysis metabolite | 84 | 35 | 2 | 121 | Norman *et al.*, 2021 |
| ADB-FUBINACA | 7 | 0 | 0 | 7 | Norman *et al.*, 2021 |
| MDMB-4en-PINACA | 0 | 23 | 142 | 165 | Norman *et al.*, 2021 |
| 4F-MDMB-BICA | 0 | 0 | 17 | 17 | Norman *et al.*, 2021 |
| 5F-ABICA amide hydrolysis metabolite | 0 | 1 | 21 | 22 | Norman *et al.*, 2021 |
| 5F-MDMB-PICA | 28 | 168 | 180 | 376 | Norman *et al.*, 2021 |
| AMB-CHMICA | 1 | 0 | 0 | 1 | Norman *et al.*, 2021 |
| AMB-FUBICA | 0 | 1 | 0 | 1 | Norman *et al.*, 2021 |
| AMB-4en-PICA | 0 | 0 | 1 | 1 | Norman *et al.*, 2021 |
| **Germany** | **2019** |  |  | **Tot** | **Reference** |
| 5F-Cumyl-PEGACLONE and metabolites | 1 | No data | No data | 1 | Giorgett *et al., 2019* |
| **England** | **2014/2015** | **2015** |  | **Tot** | **Reference** |
| 5F-AB-PINACA | 1 | 0 | No data | 1 | Bond and Hudson, 2015 |
| 5F-ADB-PINACA | 1 | 1 | No data | 2 | Bond and Hudson, 2015 |
| 5F-AKB-48 | 1177 | 272 | No data | 1449 | Bond and Hudson, 2015 |
| 5F-PB-22 | 24 | 7 | No data | 31 | Bond and Hudson, 2015 |
| 5F-UR-144 | 80 | 6 | No data | 86 | Bond and Hudson, 2015 |
| AB-FUBINACA | 3 | 0 | No data | 3 | Bond and Hudson, 2015 |
| AKB-48 | 3 | 3 | No data | 6 | Bond and Hudson, 2015 |
| AM-2201 | 87 | 13 | No data | 100 | Bond and Hudson, 2015 |
| AM-694 | 18 | 0 | No data | 18 | Bond and Hudson, 2015 |
| Cumyl-5F-PINACA | 13 | 9 | No data | 22 | Bond and Hudson, 2015 |
| MAM-2201 | 42 | 1 | No data | 43 | Bond and Hudson, 2015 |
| MDMB-CHMICA | 371 | 210 | No data | 581 | Bond and Hudson, 2015 |
| STS-135 | 9 | 0 | No data | 9 | Bond and Hudson, 2015 |
| THJ-018 | 3 | 0 | No data | 3 | Bond and Hudson, 2015 |
| THJ-2201 | 9 | 0 | No data | 9 | Bond and Hudson, 2015 |
| UR-144 | 72 | 1 | No data | 73 | Bond and Hudson, 2015 |
| **Engalnd** | **N/A** |  |  | **Tot** | **Reference** |
| MDMB-CHMICA | 3 | No data | No data | 3 | Meyappan *et al.*, 2016 |
| **Norway** | **N/A** |  |  | **Tot** | **Reference** |
| AM-2201 | 9 | No data | No data | 9 | Øiestad *et al., 2013* |
| JWH-018 | 9 | No data | No data | 9 | Øiestad *et al., 2013* |
| **USA** | **2019** |  |  | **Tot** | **Reference** |
| 4F-MDMB-BINACA 3,3-dimethylbutanoic acid | 3 | No data | No data | 3 | Norman *et al.*, 2021 |
| 5F-MDMB-PICA 3,3-dimethylbutanoic acid | 11 | No data | No data | 11 | Norman *et al.*, 2021 |
| **USA** | **2019** |  |  | **Tot** | **Reference** |
| MDMB-4en-PINACA 3,3-dimethylbutanoic acid | 1 | No data | No data | 1 | Kroutulski *et al. 2020* |
| **USA** | **2018/2019** |  |  | **Tot** | **Reference** |
| 5F-AMB butanoic acid conjugated metabolite | 2 | No data | No data | 2 | Hvozdovich *et al., 2019* |
| 5F-MDMB-PINACA aka 5F-ADB butanoic acid conjugated metabolite | 52 | No data | No data | 52 | Hvozdovich *et al., 2019* |
| AB-CHMINACA butanoic acid conjugated metabolite | 1 | No data | No data | 1 | Hvozdovich *et al., 2019* |
| FUB-AMB butanoic acid conjugated metabolite | 21 | No data | No data | 21 | Hvozdovich *et al., 2019* |
| MDMB-FUBINACA butanoic acid conjugated metabolite | 2 | No data | No data | 2 | Hvozdovich *et al., 2019* |
| **USA** | **N/A** |  |  | **Tot** | **Reference** |
| 4F-MDMB-BINACA | 1 | No data | No data | 1 | Kleis *et. al.*, 2020 |
| 5F-MDMB-PICA | 1 | No data | No data | 1 | Kleis *et. al.*, 2020 |
| **USA** | **N/A** |  |  | **Tot** | **Reference** |
| ADB-FUBINACA | 1 | No data | No data | 1 | Nacca *et al., 2018* |

Table 4. Common, street and IUPAC names of NPS mentioned in the review

| **Common name** | **Street name** | **Systematic name** |
| --- | --- | --- |
| 1-benzylpiperazine | BZP and A2 | 1-benzylpiperazine |
| 4-methyl-methamphetamine | 4-MMA | *N*-methyl-1-(4-methylphenyl)propan-2-amine |
| 4F-MDMB-BICA (aka 4F-MDMB-BUTICA) | Spice and black mamba | Methyl (S)-2-(1-(4-fluorobutyl)- 1H-indole-3-carboxamido)-3,3- dimethylbutanoate |
| 4F-MDMB-BINACA (aka 4F-MDMB-BUTINACA) | Spice and black mamba | Methyl (2R)-2-{[1-(4- fluorobutyl)-1H-indazole-3-carbonyl]amino}-3,3-dimethylbutanoate |
| 4F-PHP | Bath salts | 1-(4-fluorophenyl)-2-(pyrrolidin-1-yl)hexan-1-one, monohydrochloride |
| 5F-AB-PINACA | Spice and black mamba | *N*-[(1S)-1-(aminocarbonyl)-2-methylpropyl]-1-(5-fluoro)pentyl-1H-indazole-3-carboxamide |
| 5F-ADB (aka 5F-MDMB-PINACA) | Spice and black mamba | Methyl-[2-(1-(5-fluoropentyl)-1H-indazole-3-carboxamido)-3,3-dimethylbutanoate] |
| 5F-ADB-PINACA | Spice and black mamba | *N*-(1-amino-3,3-dimethyl-1-oxobutan-2-yl)-1-(5-fluoropentyl)-1H-indazole-3-carboxamide |
| 5F-AMB (aka 5F-MMB-PINACA aka 5F-AMB-PINACA) | Spice and black mamba | Methyl 2-({[1-(5-fluoropentyl)-1H-indazol-3-yl]carbonyl}amino)-3-methylbutanoate |
| 5F-APINACA (aka 5F-AKB-48) | Spice and black mamba | *N*-(1-adamantyl)-1-(5-fluoropentyl)-1H-indazole-3 -carboxamide |
| 5F-Cumyl-PEGACLONE | Spice and black mamba | 5-(5-fluoropentyl)-2-(1-methyl-1-phenylethyl)-pyrido[4,3-b]indol-1-one |
| 5F-Cumyl-PICA | Spice and black mamba | 1-(5-fluoropentyl)-N-(1-methyl-1-phenylethyl)-1H-indole-3-carboxamide |
| 5F-Cumyl-PINACA | Spice and black mamba | 1-(5-fluoropentyl)-N-(1-methyl-1-phenylethyl)-1H-indazole-3-carboxamide |
| 5F-EMB-PICA (aka EMB-2201) | Spice and black mamba | Ethyl (1-(5-fluoropentyl)-1H-indole-3-carbonyl)-L-valinate |
| 5F-MDMB-P7AICA | Spice and black mamba | Methyl 2-{[1-(5-fluoropentyl)-1H-pyrrolo[2,3-b]pyridin-3-yl]formamido}-3,3-dimethylbutanoate |
| 5F-MDMB-PICA | Spice and black mamba | Methyl 2-[[1-(5-fluoropentyl)indole-3-carbonyl]amino]-3,3-dimethyl-butanoate |
| 5F-MPP-PICA (aka MPHP-2201) | Spice and black mamba | Methyl (1-(5-fluoropentyl)- 1H-indole-3-carbonyl)-L-phenylalaninate |
| 5F-PB-22 | Spice and black mamba | Quinolin-8-yl 1-(5-fluoropentyl)-1H-indole-3-carboxylate |
| 5F-UR-144 (aka XLR-11) | Spice and black mamba | (1-( 5-fluoropentyl)-1H-indol-3-yl)(2,2,3,3-tetramethylcyclopropyl)methanone |
| AB-CHMINACA | Spice and black mamba | *N*-[(1S)-1-(aminocarbonyl)-2-methylpropyl]-1-(cyclohexylmethyl)-1H-indazole-3-carboxamide |
| AB-FUBINACA | Spice and black mamba | *N*-(1-amino-3-methyl-1-oxobutan-2-yl)-1-(4-fluorobenzyl)-1H-indazole-3-carboxamide |
| Acryloylfentanyl | N/A | *N*-(1-phenethylpiperidin-4-yl)-N-phenylacrylamide |
| ADB-BINACA | Spice and black mamba | *N*-(1-amino-3,3-dimethyl-1-oxobutan-2-yl)-1-benzyl-1H-indazole-3-carboxamide |
| ADB-CHMINACA (aka MAB-CHMINACA) | Spice and black mamba | *N*-[1-(aminocarbonyl)-2,2-dimethylpropyl]-1-(cyclohexylmethyl)-1H-indazole-3-carboxamide |
| ADB-FUBINACA | Spice and black mamba | *N*-[(1S)-1-(aminocarbonyl)-2,2-dimethylpropyl]-1-[(4-fluorophenyl)methyl]-1H-indazole-3-carboxamide |
| AM-2201 | Spice and black mamba | 1-[(5-fluoropentyl)-1H-indol-3-yl]-(naphthalen-1-yl)methanone |
| AM-694 | Spice and black mamba | 1-[(5-fluoropentyl)-1H-indol-3-yl]-(2-iodophenyl)methanone |
| AMB-4en-PICA (aka MMB-4en-PICA, MMB022) | Spice and black mamba | Methyl (1-(pent-4-en-1-yl)-1H-indole-3-carbonyl)-L-valinate |
| AMB-FUBICA | Spice and black mamba | Methyl 2-[[1-[(4-fluorophenyl)methyl]indole-3-carbonyl]amino]-3-methyl-butanoate |
| AMB-FUBINACA (aka FUB-AMB) | Spice and black mamba | Methyl-2-(1-(4-fluorobenzyl)-1H-indazole-3-carboxamide)-3-methylbutanoate |
| APINACA (aka AKB-48) | Spice and black mamba | *N*-(1-adamantyl)-1-pentyl-1H-indazole-3-carboxamide |
| Carfentanil | N/A | Methyl 1-phenethyl-4-(*N*-phenylpropionamido)piperidine-4-carboxylate |
| Cumyl-4CN-B7AICA | Spice and black mamba | 1-(4-cyanobutyl)-N-(2-phenylpropan-2-yl)-1H-pyrrolo[2,3-b]pyridin-3-carboxamide |
| Cumyl-4CN-BINACA (aka Cumyl-CYBINACA aka SGT-78) | Spice and black mamba | 1-(4-cyanobutyl)-N-(1-methyl-1-phenyl-ethyl)indazole-3-carboxamide |
| Cumyl-5F-PINACA (aka SGT-25) | Spice and black mamba | 1-(5-fluoropentyl)-N-(1-methyl-1-phenylethyl)-1H-indazole-3-carboxamide |
| Cumyl-CB-MeGACLONE | Spice and black mamba | 5-(cyclobutylmethyl)-2-(2-phenylpropan-2-yl)-2,5-dihydro-1H-pyrido[4,3-b]indol-1-one |
| Cumyl-CBMICA | Spice and black mamba | 1-(cyclobutylmethyl)-N-(2-phenylpropan-2-yl)-1H-indole-3-carboxamide |
| Cumyl-CBMINACA | Spice and black mamba | 1-(cyclobutylmethyl)-N-(2-phenylpropan-2-yl)-1H-indazole-3-carboxamide |
| CUMYL-PEGACLONE (aka SGT-151) | Spice and black mamba | 5-pentyl-2-(2-phenylpropan-2-yl)pyrido[4,3-b]indol-1-one |
| Cumyl‐PeGACLONE (aka SGT-151) | Spice and black mamba | 5-(5-fluoropentyl)-2-(1-methyl-1-phenylethyl)-pyrido[4,3-b]indol-1-one |
| Cyclopropylfentanyl | N/A | *N*-phenyl-N-[1-(2-phenylethyl)piperidin-4-yl]cyclopropanecarboxamide |
| EG-018 | Spice and black mamba | Naphthalen-1-yl(9-pentyl-9H-carbazol-3-yl)methanone |
| Ethylphenidate | Nopaine and Fake cocaine | Ethyl 2-phenyl-2-piperidin-2-ylacetate |
| Etizolam | Street Valium | 4-(2-chlorophenyl)-2-ethyl-9-methyl-6H-thieno[3,2-f][1,2,4]triazolo[4,3-a][1,4]diazepine |
| FUB-144 (aka FUB-UR-144) | Spice and black mamba | [1-[(4-fluorophenyl)methyl]indol-3-yl]-(2,2,3,3-tetramethylcyclopropyl)methanone |
| FUB-APINACA (aka FUB-AKB48, AFB-48, AFUBINACA, FUB-APINACA) | Spice and black mamba | *N*-((3s,5s,7s)-adamantan-1-yl)-1-(4-fluorobenzyl)-1H-indazole-3-carboxamide |
| FUB-PB-22 (aka QUFUBIC) | Spice and black mamba | Quinolin-8-yl-1-(4-fluorobensyl)-1H-indole-3-carboxylate |
| JWH-018 | Spice and black mamba | Naphthalen-1-yl-(1-pentylindol-3-yl)methanon |
| JWH-081 | Spice and black mamba | (1-pentyl-3-(4-methoxy-1-naphthoyl)indole) |
| JWH-122 | Spice and black mamba | 1-pentyl-3-(4-methyl-1-naphthoyl)indole |
| JWH-210 | Spice and black mamba | 1-pentyl-3-(4-ethyl-1-naphthoyl)indole |
| Kava | Kava kava and kawa | Piper methysticum |
| MAM-2201 | Spice and black mamba | 1-(5-fluoropentyl)-3-(4-methyl-naphthoyl)indole |
| MDMB-4en-PINACA | Spice and black mamba | Methyl (S)-3,3-dimethyl-2-(1-(pent-4-en-1-yl)-1H-indazole-3-carboxamido)butanoate |
| MDMB-CHMICA (aka MDMB-CHMINACA) | Spice and black mamba | *N*-[[1-(cyclohexylmethyl)-1H-indol-3-yl]carbonyl]-3-methyl-valine, methyl ester |
| MDMB-FUBINACA (aka FUB-MDMB aka MDMB-Bz-F) | Spice and black mamba | 2-[[1-[(4-fluorophenyl) methyl] indazole-3-carbonyl] amino]-3,3-dimethyl-butanoate |
| Mephedrone (aka 4-methyl methcathinone) | Bath salts, M-CAT and Meow Meow | (RS)-2-methylamino-1-(4-methylphenyl)propan-1-one |
| Methiopropamine | MPA | *N*-Methyl-1-(thiophen-2-yl)propan-2-amine |
| Methoxphenidine | MXP | 1-(1-(2-methoxyphenyl)-2-phenylethyl)piperidine, monohydrochloride |
| Methylethcathinone | Bath salts and 2-MEC | 2-(ethylamino)-1-(2-methylphenyl)propan-1-one |
| Methylhexaneamine | DMMA | 4-methylhexan-2-amine |
| Methylone | Bath salts and MDM-CAT | 1-(1,3-Benzodioxol-5-yl)-2-(methylamino)propan-1-one |
| Methylphenidate | Nopaine and fake cocaine | Methyl 2-phenyl-2-piperidin-2-ylacetate |
| MMB-CHMICA (aka AMB-CHMICA) | Spice and black mamba | Methyl 2-(1-(cyclohexylmethyl)-1H-indazole-3-carboxamide)-3-methylbutanoate |
| MMB‐2201 | Spice and black mamba | Methyl (1-(5-fluoropentyl)-1H-indole-3-carbonyl)valinate |
| PB-22 (aka QUPIC) | Spice and black mamba | 1-Pentyl-1*H*-indole-3-carboxylic acid 8-quinolinyl ester |
| QUCHIC (aka BB-22) | Spice and black mamba | Quinolin-8-yl 1-(cyclohexylmethyl)-1H-indole-3-carboxylate |
| (R)-4F-MDMB-BINACA | Spice and black mamba | Methyl (2R)-2-{[1-(4-fluorobutyl)-1H-indazole-3- carbonyl]amino}-3,3-dimethylbutanoate |
| (R)-5F-ADB (aka (R)-5F-MDMB-PINACA) | Spice and black mamba | Methyl-[2R-(1-(5-fluoropentyl)-1H-indazole-3-carboxamido)-3,3-dimethylbutanoate] |
| (R)-5F-MDMB-PICA | Spice and black mamba | Methyl-(2R)-[[1-(5-fluoropentyl)indole-3-carbonyl]amino]-3,3-dimethyl-butanoate |
| (R)-MDMB-4en-PINACA | Spice and black mamba | Methyl (R)-3,3-dimethyl-2-(1-(pent-4-en-1-yl)-1H-indazole-3-carboxamido)butanoate |
| STS-135 (aka 5F-APICA) | Spice and black mamba | 1-(5-fluoropentyl)-N-tricyclo[3.3.1.13,7]dec-1-yl-1H-indole-3-carboxamide |
| THJ-018 | Spice and black mamba | Naphthalen-1-yl(1-pentyl-1H-indazol-3-yl)methanone |
| THJ-2201 | Spice and black mamba | (1-(5-fluoropentyl)-1H-indazol-3-yl)(naphthalen-1-yl)methanone |
| UR-144 | Spice and black mamba | (1-pentyl-1H-indol-3-yl)(2,2,3,3-tetramethylcyclopropyl)methanone |

Figure SI1. Trends of scientific publications of NPS reported in prison settings from 1978-2020

Figure SI2. Routes in which NPS are smuggled into prison

Figure SI3. Forms in which NPS are smuggled into prison
